# Adverse Pregnancy Outcomes and International Immigration Status: A Systematic Review and Meta-analysis

**DOI:** 10.5334/aogh.3591

**Published:** 2022-06-28

**Authors:** Samira Behboudi-Gandevani, Razieh Bidhendi-Yarandi, Mohammad Hossein Panahi, Abbas Mardani, Piret Paal, Christina Prinds, Mojtaba Vaismoradi

**Affiliations:** 1 Faculty of Nursing and Health Sciences, Nord University, 8049 Bodø, Norway; 2 Department of Biostatistics, University of Social Welfare and Rehabilitation Sciences, Tehran, Iran; 3 Department of Epidemiology, School of Public Health and Safety, Shahid Beheshti University of Medical Sciences, Tehran, Iran; 4 Nursing Care Research Center, Department of Medical Surgical Nursing, School of Nursing and Midwifery, Iran University of Medical Sciences, Tehran, Iran; 5 Institute of Nursing Science and Practice, Paracelsus Medical University, A-5020 Salzburg, Austria; 6 Department of Clinical Research, University South Denmark, 5230 Odense, Denmark; 7 Department of Midwifery Education, University College South Denmark, Esbjerg, Denmark; 8 Faculty of Science and Health, Charles Sturt University, Orange NSW 2800, Australia

**Keywords:** Adverse Pregnancy Outcomes, International immigration, Meta-analysis

## Abstract

**Background:**

Disparities in health outcomes between immigrant and native-origin populations, particularly pregnant women, pose significant challenges to healthcare systems. The aim of this systematic-review and meta-analysis was to investigate the risk of adverse pregnancy outcomes among immigrant-women compared to native-origin women in the host country.

**Methods:**

PubMed (including MEDLINE), Scopus, and Web of Science were searched to retrieve studies published in English language up to September 2020. All observational studies examining the prevalence of at least one of the short-term single pregnancy outcomes for immigrants who crossed international borders compared to native-origin pregnant population were included. The meta-prop method was used for the pooled-estimation of adverse pregnancy-outcomes’ prevalence. For pool-effect estimates, the association between the immigration-status and outcomes of interest, the random-effects model was applied using the model described by DerSimonian and Laird. I^2^ statistic was used to assess heterogeneity. The publication bias was assessed using the Harbord-test. Meta-regression was performed to explore the effect of geographical region as the heterogeneity source.

**Findings:**

This review involved 11 320 674 pregnant women with an immigration-background and 56 102 698 pregnant women as the native-origin population. The risk of emergency cesarean section (Pooled-OR = 1.1, 95%CI = 1.0–1.2), shoulder dystocia (Pooled-OR = 1.1, 95%CI = 1.0–1.3), gestational diabetes mellites (Pooled-OR = 1.4, 95%CI = 1.2–1.6), small for gestational age (Pooled-OR=1.3, 95%CI = 1.1–0.4), 5-min Apgar less than 7 (Pooled-OR = 1.2, 95%CI = 1.0–1.3) and oligohydramnios (Pooled-OR = 1.8, 95%CI = 1.0–3.3) in the immigrant women were significantly higher than those with the native origin background. The immigrant women had a lower risk of labor induction (Pooled-OR = 0.8, 95%CI = 0.7–0.8), pregnancy induced hypertension (Pooled-OR = 0.6, 95%CI = 0.5–0.7) preeclampsia (Pooled-OR = 0.7, 95%CI = 0.6–0.8), macrosomia (Pooled-OR = 0.8, 95%CI = 0.7–0.9) and large for gestational age (Pooled-OR = 0.8, 95%CI = 0.7–0.8). Also, the risk of total and primary cesarean section, instrumental-delivery, preterm-birth, and birth-trauma were similar in both groups. According to meta-regression analyses, the reported ORs were not influenced by the country of origin.

**Conclusion:**

The relationship between the immigration status and adverse perinatal outcomes indicated a heterogenous pattern, but the immigrant women were at an increased risk of some important adverse pregnancy outcomes. Population-based studies with a focus on the various aspects of this phenomena are required to explain the source of these heterogenicities.

## Introduction

The immigrant population has been defined as any person moving across an international border, regardless of the person’s legal status; whether it is voluntary or involuntary and what the causes of movement are; or what the length of the stay is [1]. This population constitutes a heterogeneous and wide category group including refugees, asylum seekers, illegal and undocumented immigrants, economic and transient immigrants. It is a hallmark of global development over the last millennia [2]. It has been estimated that more than 3.5% of the world’s population are immigrants in the world in 2020, and immigration has increased dramatically over the past two decades [1]. The underlying reasons for immigration are multifactorial, involving a complex interaction between factors within and beyond individuals’ control including political, socioeconomic, and educational, along with more acute drivers such as natural disasters, violence, and conflict [3].

Health manifestation of immigrants reflect their past medical histories, the disease burden and quality of care in the original and host countries [4]. Although there is a heterogeneity in the degree to which immigrants are vulnerable to inadequate health care, they have been identified as generally vulnerable population. These differences are more complicated by pregnancy. Subsequently, a specific attention should be paid to pregnant women. Interactions between socio-material deprivation factors aggravated by stressors involved in the immigration process can play an important role [5]. However, the results of studies on the adverse perinatal health of immigrants are conflicting [4]. Some studies reported that the perinatal health of immigrants were equal or better compared to the native origin population. In contrast, some studies showed that the risk of adverse pregnancy outcomes were poorer than women in the host country [6,7,8,9,10,11]. Given the lack of conclusive evidence, the aim of this systematic review and meta-analysis was to investigate the risk of adverse maternal and neonatal outcomes in immigrant women compared to native-origin women in the host country.

## Methodology

The research protocol for this systematic review and meta-analysis was developed before the study and was used as the guideline to conduct this research (Supplementary Table 1). A systematic review and meta-analysis was conducted based on the Cochrane methodology for systematic review and meta-analysis studies. The research process has been informed by the guideline for the preferred reporting items for systematic reviews and meta-analyses (PRISMA) [12]. The review question was framed based on the PICO statement as follows:

P: pregnant women with the history of pregnancy and their neonates; I: international immigration; C: pregnant women with the native-origin background; O: adverse maternal and neonatal outcomes.

The research objectives were as follows:

- To study the pooled prevalence and risk of adverse maternal outcomes in pregnant women with the immigration background compared to their native-origin counterparts;
- To study the pooled prevalence and risk of adverse neonatal outcomes in pregnant women with the immigration background compared to their native-origin counterparts.

### Eligibility Criteria

Studies were identified eligible if they (1) examined immigrant women who crossed international borders; (2) reported one type of data including number, prevalence, or the risk of incidents for at least one of the short-term single maternal and neonatal outcomes; (3) compared those outcomes between pregnant women with the immigration background and the native-origin population (4) and generally without time limitation.

Exclusion criteria were non-original studies including reviews, commentaries, editorials, letters, case reports, conference proceedings, books, original studies without accurate and clear data on research variables, duplicated data, and also studies focusing on a specific minor population such as adolescents. However, no restrictions were applied on the immigrant origin, status or length of time for the receiving country.

### Search Strategy

For this systematic review and meta-analysis, online databases of PubMed (including MEDLINE), Scopus, and Web of Science that covered the main percentage of observational studies published in English were searched up to September 2020. Also, a manual search in the references lists of selected studies and other relevant reviews was carried out to maximize the identification of eligible studies.

Two review authors performed the search individually using the following key words and phrases alone or in combination as follows: (immigration OR migration OR immigrant OR migrant OR emigrant OR asylum seeker OR asylum seeking OR asylum OR refugee) AND (“adverse pregnancy outcomes” OR “pregnancy outcomes” OR “pregnancy complications” OR abortion OR miscarriage OR “pregnancy loss” OR “fetal death” OR “stillbirth” OR “preeclampsia” OR “gestational hypertension” OR PIH OR “gestational diabetes” OR hemorrhage OR “postpartum hemorrhage” OR PPH OR “Placenta abruption” OR “placenta previa” OR preterm OR “premature rupture of membrane” OR PROM OR “Intra uterine growth restriction” OR IUGR OR “Low birth weight” OR LBW OR oligohydramnios OR Apgar OR “fetal distress” OR “neonatal distress” OR RDS OR “neonatal death” OR “neonatal mortality” OR “neonatal admission” OR “NICU admission” OR malformation OR anomalies OR “birth weight” OR LGA OR “large for gestational age” OR SGA OR “small for gestational age” OR “gestational diabetes” OR GDM OR IUFD OR “intra uterine fetal death” OR cesarean OR “operative delivery” OR “instrumental delivery” OR vacuum).

### Study Selection and Data Extraction

The titles, abstracts, and full texts of studies were examined independently by two review authors to determine whether they met the inclusion criteria. The decision on the final inclusion of studies was made through reaching a consensus by all review authors through holding discussions. Selected articles meeting the inclusion criteria were included in data analysis and research synthesis.

The following data were extracted from the included studies: origin of study; publication year; study period; size of study population; population characteristics including age and body mass index (BMI); outcome measurements including the number, prevalence or risk of adverse pregnancy events. To prevent bias in the data extraction and data entry, double checking of the data extraction process was performed before meta-analysis.

### Study Outcomes

The important maternal events of labor induction, total cesarean-section (S-C), primary C-S, emergency C-S, instrumental delivery, gestational diabetes mellitus (GDM), preeclampsia, and pregnancy-induced hypertension (PIH) were considered. Also, neonatal events consisted of macrosomia, large for gestational age (LGA), small for gestational age (SGA), admission to the neonatal intensive care unit (NICU), respiratory distress syndrome (RDS), Apgar scores less than 7 at five minutes, shoulder dystopia, birth trauma, oligohydramnios, and preterm birth.

### Quality Appraisal and Statistical Data Analysis

Selected studies (n = 126) were appraised with regard to the quality of their methodological structures and the presentation of results. Three reviewers who were blind to the study authors, institution, and journal name, assessed the quality of each these studies individually. Observational studies including cross-sectional, case–control, and cohort were appraised using the Newcastle–Ottawa scale [13]. Three domains were scored for the selection and comparability of study cohorts, and to determine the outcome of interest. Studies with scores above 6 were judged high quality, 4–6 moderate quality, and less than 4 low quality.

The meta-prop method helped with the pooled estimation of the prevalence of adverse pregnancy outcomes. To study the association between the immigration status and the outcomes of interest, pooled odds ratio (OR) (with 95% CI) was considered the effect size. The random-effects model described by DerSimonian and Laird was used for the data analysis. Corresponding forest plots were constructed for both the pooled prevalence and odds ratio (OR) of the outcomes. Study heterogeneity was assessed using the inconsistency index (I^2^-statistic) and > 50% was considered substantial heterogeneity. Publication bias was assessed through the Harbord test. Meta-regression was performed to explore the association between maternal age and the risk of adverse pregnancy outcomes in immigrant women compared to those with the native origin background. Moreover, we assessed the effect of geographical region as the source of heterogeneity. P < 0.05 was set as the significance level. Data analyses were conducted using Stata (version 14; STATA Inc., College Station, TX, USA).

## Results

### Search Results, Study Selection, Study Characteristics, and Quality Appraisal

**Figure 1:**
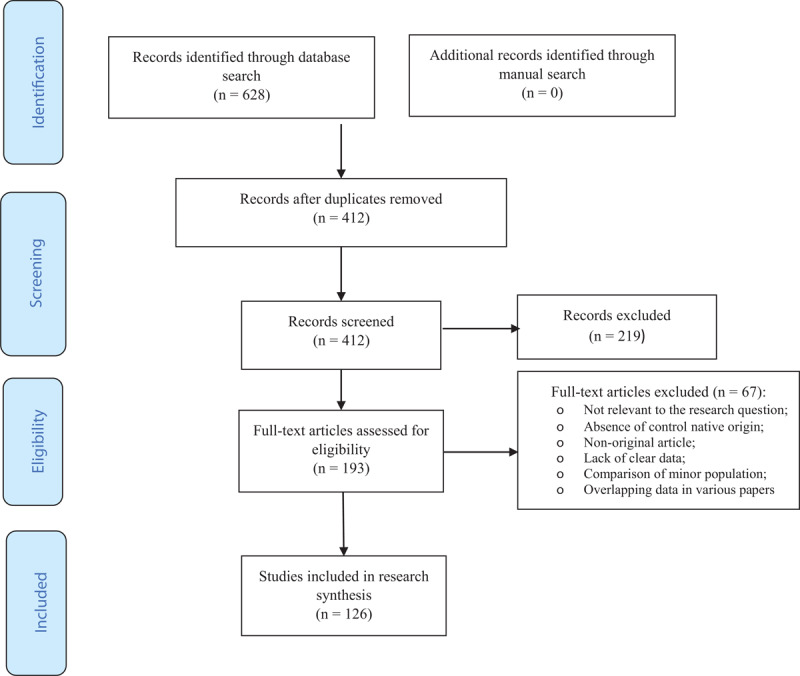
The PRISMA flowchart for the search process.

The literature search yielded 628 studies. They were saved in the EndNote library and 216 duplicates were identified. The remaining 412 studies underwent the review process. Of these, 219 were excluded after title and abstract screening. Full text of 193 studies were read and appraised and a total of 126 studies met the inclusion criteria, involving 11 320 674 pregnant women with the immigration background and 56 102 698 native origin women (Figure 1). Characteristics of these studies have been summarized in supplementary Table 2. The quality appraisal of the included studies has been presented in supplementary Tables 3 and 4. A total of 106 studies were judged as high quality [11,14,15,16,17,18,19,20,21,22,23,24,25,26,27,28,29,30,31,32,33,34,35,36,37,38,39,40,41,42,43,44,45,46,47,48,49,50,51,52,53,54,55,56,57,58,59,60,61,62,63,64,65,66,67,68,69,70,71,72,73,74,75,76,77,78,79,80,81,82,83,84,85,86,87,88,89,90,91,92,93,94,95,96,97,98,99,100,101,102,103,104,105,106,107,108,109,110,111,112,113,114,115,116,117,118], 20 as moderate quality [119,120,121,122,123,124,125,126,127,128,129,130,131,132,133,134,135,136,137,138], and no study had low quality.

A total of 21 studies (17%) were conducted in the USA and Canada [25,37,38,44,47,49,53,72,75,77,84,86,89,91,96,100,106,109,116,118,124], 73 studies (58%) in Europe [11,15,17,19,23,24,27,28,29,30,33,34,35,36,39,40,41,42,43,45,46,47,51,54,55,56,57,58,59,63,64,65,66,67,69,70,73,74,76,79,80,81,82,83,85,90,92,93,94,95,97,98,99,102,103,104,105,107,108,110,113,114,115,117,122,126,130,131,132,133,136,138], 19 studies (15%) in Asia [16,22,26,31,50,52,71,78,87,101,119,120,121,123,125,127,129,134,135], 3 studies (2%) in South America [112,128,137] and 10 studies (8%) in Australia and New Zealand [14,18,20,21,32,60,61,62,68,88].

### Meta-analysis and Meta-regression

Compared to those with the native origin background, the immigrant women were more likely to be younger, although comparison was not statistically significant [pooled mean (CI 95%): 29.9 (29.9) versus 29.2 (29.1) years (P < 0.095)]. The pooled prevalence of adverse maternal and neonatal outcomes among the immigrant and native origin populations have been presented in Figure 2 and Supplementary Figures 1–17.

**Figure 2:**
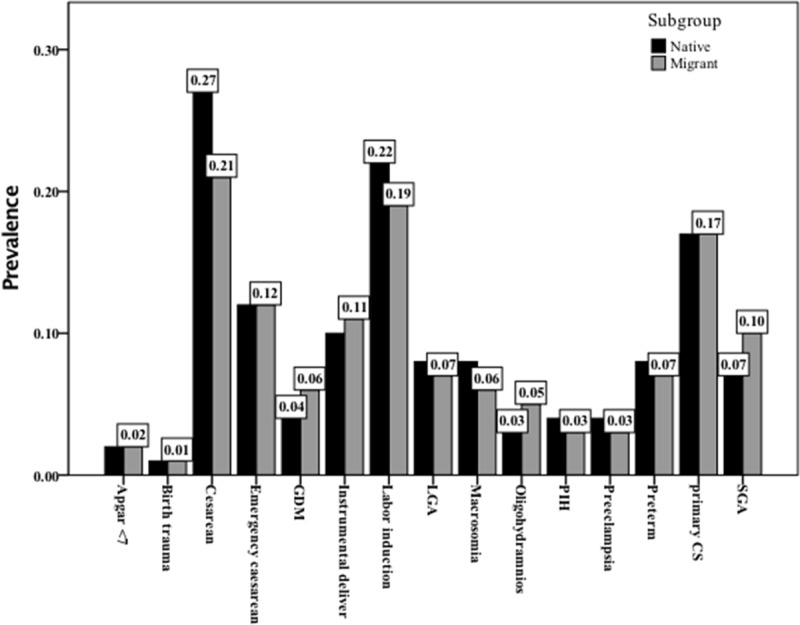
Pooled prevalence of adverse maternal and neonatal outcomes in the studied population including immigrant women and those with the native origin background. GDM: gestational diabetes mellitus, LGA: large for gestational diabetes, PIH: pregnancy induced hypertension, SGA: small for gestational age.

Table 1 outlines the prevalence and pooled ORs of adverse maternal and neonatal outcomes, and estimation of heterogeneity and publication bias among the pregnant women with the immigration background compared to their native origin counterparts. No substantial publication bias based on the Harbord test was observed (Table 1), except for GDM, which was corrected using the trim and fill method of publication bias adjustment.

**Table 1:** Heterogeneity, estimation of publication bias, and meta-analysis of the studies on the risk of adverse maternal and neonatal outcomes among immigrant women and native origin women.


OUTCOME	SAMPLE SIZE OF IMMIGRANT		SAMPLE SIZE OF NATIVE ORIGIN		POOLED PREVALENCE (95% CI)*		PUBLICATION BIAS HARBORD TEST*	HETEROGENEITY I-SQUARED %	POOLED OR (95% CI)
		
	EVENT	TOTAL	EVENT	TOTAL	MIGRANT	NATIVE ORIGIN			

**Adverse maternal outcome**									

Cesarean section	148931	660205	3677824	20596518	0.21 (0.20, 0.23)	0.27 (0.25, 0.29)	0.739	**98.1**	0.992 (0.941, 1.045)

Emergency cesarean section	50600	395162	993568	9277701	0.12 (0.11, 0.13)	0.11 (0.10, 0.13)	0.466	**97.2**	**1.129 (1.048, 1.215)**

Primary cesarean section	6279	44318	539154	2788776	0.17 (0.15, 0.19)	0.17 (0.14, 0.20)	0.755	**94.1**	0.915 (0.788, 1.062)

Labor induction	525115	291182	1335153	5634534	0.19 (0.18, 0.21)	0.22 (0.19, 0.25)	0.484	**93.7**	**0.837 (0.793, 0.883)**

Instrumental delivery	51680	456979	1124217	11320234	0.11 (0.10, 0.12)	0.10 (0.09, 0.12)	0.575	**96.7**	1.027 (0.961, 1.097)

Pregnancy induced hypertension	46096	2393549	1020186	26900498	0.03 (0.02, 0.03)	0.04 (0.03, 0.05)	0.496	**98.2**	**0.663 (0.596, 0.738)**

Preeclampsia	21055	2369038	722645	27941572	0.03 (0.02, 0.03)	0.04 (0.03, 0.05)	0.156	**93.3**	**0.746 (0.692, 0.804)**

GDM	87936	2571075	982427	40242084	0.06 (0.05, 0.06)	0.04 (0.04, 0.05)	0.000*	**99.5**	**^$^1.441 (1.268, 1.636)**

**Adverse neonatal outcome**									

Macrosomia	62872	803186	1145835	13748134	0.06 (0.05, 0.07)	0.08 (0.06, 0.10)	0.263	**99.2**	**0.822 (0.721, 0.937)**

LGA	51608	663665	1252440	15590983	0.07 (0.06, 0.08)	0.08 (0.06, 0.09)	0.665	**98.9**	**0.809 (0.730, 0.898)**

SGA	162335	2987996	2435087	36623165	0.10 (0.09, 0.10)	0.07 (0.06, 0.08)	0.072	**99.3**	**1.347 (1.243, 1.460)**

5-min Apgar score less than 7	19500	1231886	449914	32466089	0.19 (0.18, 0.21)	0.02 (0.01, 0.02)	0.096	**97.5**	**1.222 (1.096, 1.362)**

Shoulder dystocia	4654	29309	59306	472459	0.21 (0.09, 0.40)	0.29 (0.03, 0.52)	0.250	**90.3**	**1.160 (1.00, 1.350)**

Preterm birth	629485	8136358	2704204	39495424	0.07 (0.06, 0.07)	0.08 (0.07, 0.09)	0.208	**93.7**	0.939 (0.913, 1.966)

Birth trauma	3353	456327	65637	5696997	0.01 (0.01, 0.02)	0.01 (0.00, 0.02)	0.893	**66.3**	0.968 (0.899, 1.043)

Oligohydramnios	825	22043	4903	243412	0.05 (0.04, 0.06)	0.03 (0.02, 0.04)	0.405	**97.7**	**1.862 (1.032, 3.360)**


GDM: Gestational diabetes; LGA: Large for gestational age; SGA: Small for gestational age; NICU: Neonatal intensive care Unit; RDS: Respiratory distress syndrome. Bold values indicate statistical significance. * Obtained from the trim and fill method of publication bias adjustment.

In term of maternal outcomes, the pooled OR of emergency C-S (Pooled OR = 1.1, 95% CI = 1.0– 1.2) and shoulder dystocia (Pooled OR = 1.1, 95% CI = 1.0–1.3), and GDM (Pooled OR = 1.4, 95% CI = 1.2–1.6) in the immigrant women were significantly higher than the women with the native origin background (Figure 3A, 3B, C). In contrast, the immigrant women had a lower risk of induction of labor (Pooled OR = 0.8, 95% CI = 0.7–0.8), pregnancy induced hypertension (Pooled OR = 0.6, 95% CI = 0.5–0.7) and preeclampsia (Pooled OR = 0.7, 95% CI = 0.6–0.8) compared to their native origin counterparts (Figure 3D, E, F). However, the risks of total and primary C-S and instrumental delivery were similar in both groups (Supplementary Figures 18–20).

**Figure 3:**
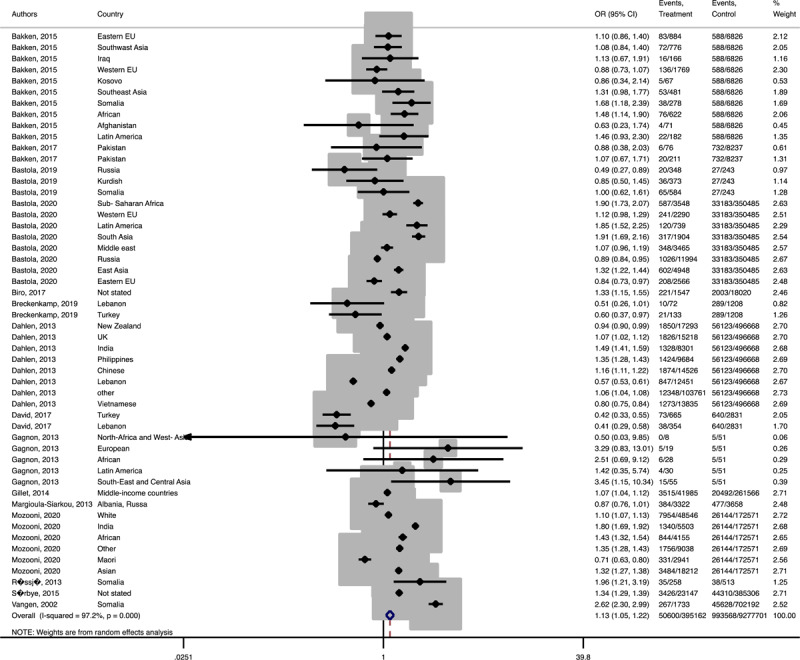
Forest plots of the pooled odds ratio of adverse maternal outcomes comparing immigrant and native origin women. **(A)** Forest plot of the pooled odds ratio of emergency cesarean section comparing immigrant and native origin women.

**Figure 3-B:**
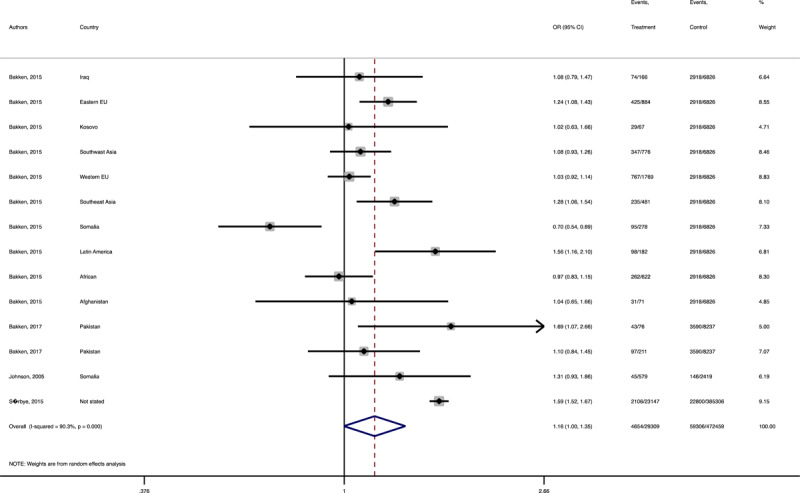
Forest plot of the pooled odds ratio of shoulder dystocia comparing immigrant and native origin women.

**Figure 3-C:**
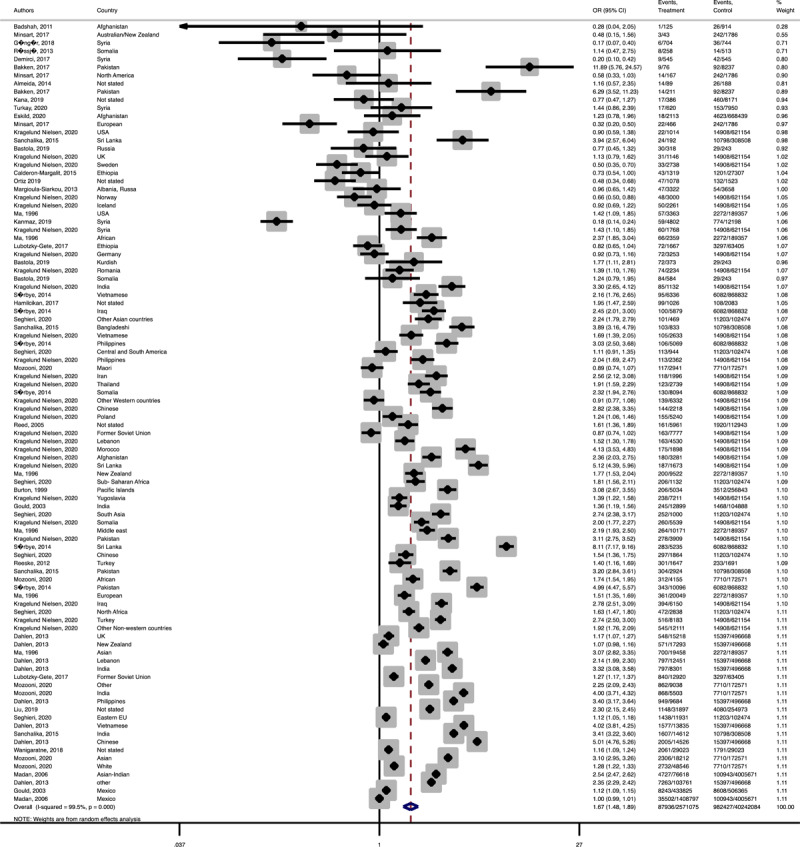
Forest plot of the pooled odds ratio of gestational diabetes mellitus comparing immigrant and native origin women.

**Figure 3-D:**
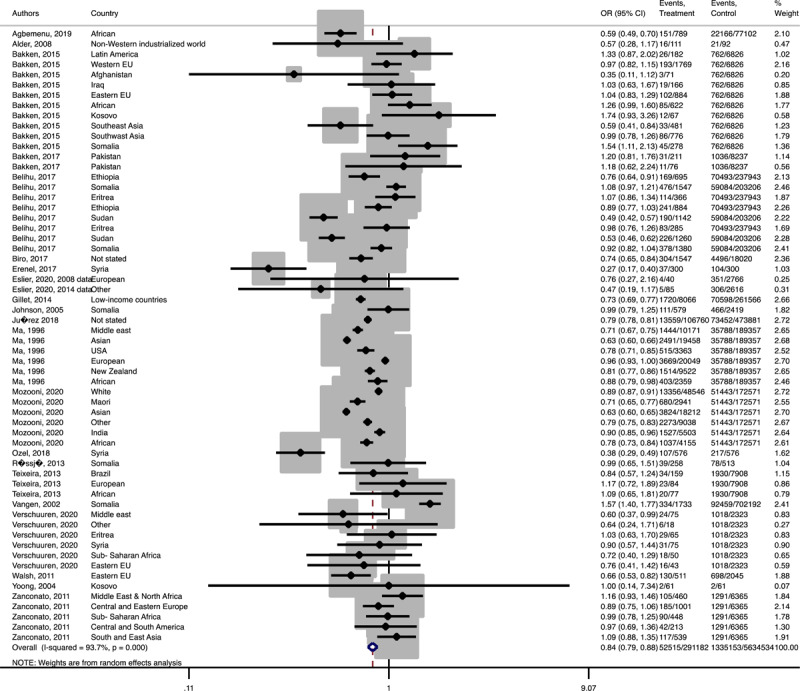
Forest plot of the pooled odds ratio of induction of labor comparing immigrant and native origin women.

**Figure 3-E:**
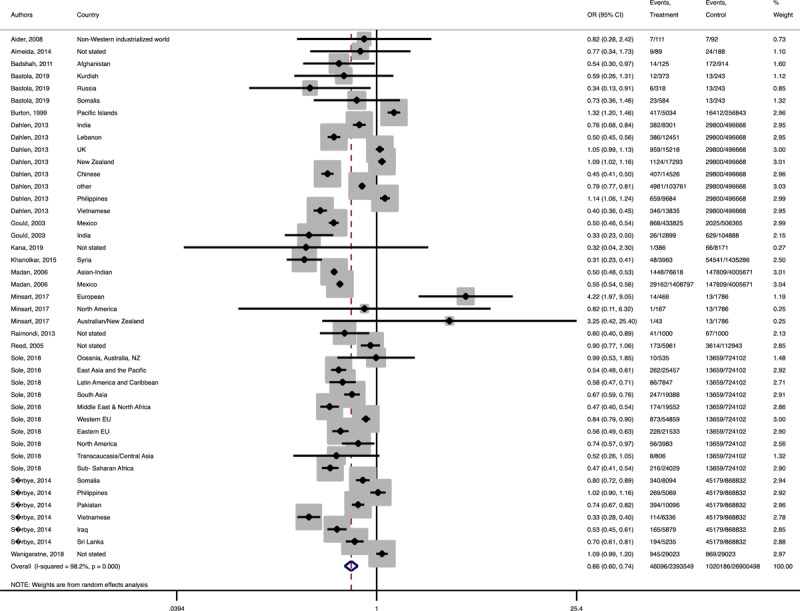
Forest plot of the pooled odds ratio of pregnancy induced hypertension comparing migrant and native origin population.

**Figure 3-F:**
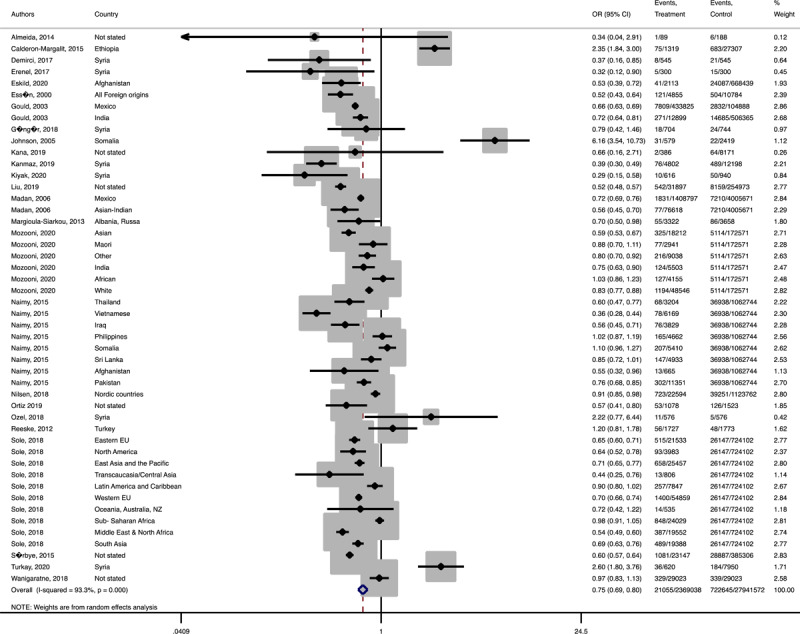
Forest plot of the pooled odds ratio of preeclampsia comparing immigrant and native origin women.

In terms of neonatal outcomes, the adverse events of preterm birth and birth trauma were not significantly different between the groups (Table 1, Supplementary Figures 21–22). However, the risk of SGA (Pooled OR = 1.3, 95% CI = 1.1–0.4), 5 min-Apgar less than 7 (Pooled OR = 1.2, 95% CI = 1.0–1.3), and oligohydramnios (Pooled OR = 1.8, 95% CI = 1.0–3.3) among the immigrant women were significantly higher than the women with the native origin background (Table 1, Figures 4A, B, C). Additionally, the immigrant women had significantly lower risk of macrosomia (Pooled OR = 0.8, 95% CI = 0.7–0.9) and LGA (Pooled OR = 0.8, 95% CI = 0.7–0.8) compared to the native origin women (Table 1, Figures 4D and E).

**Figure 4:**
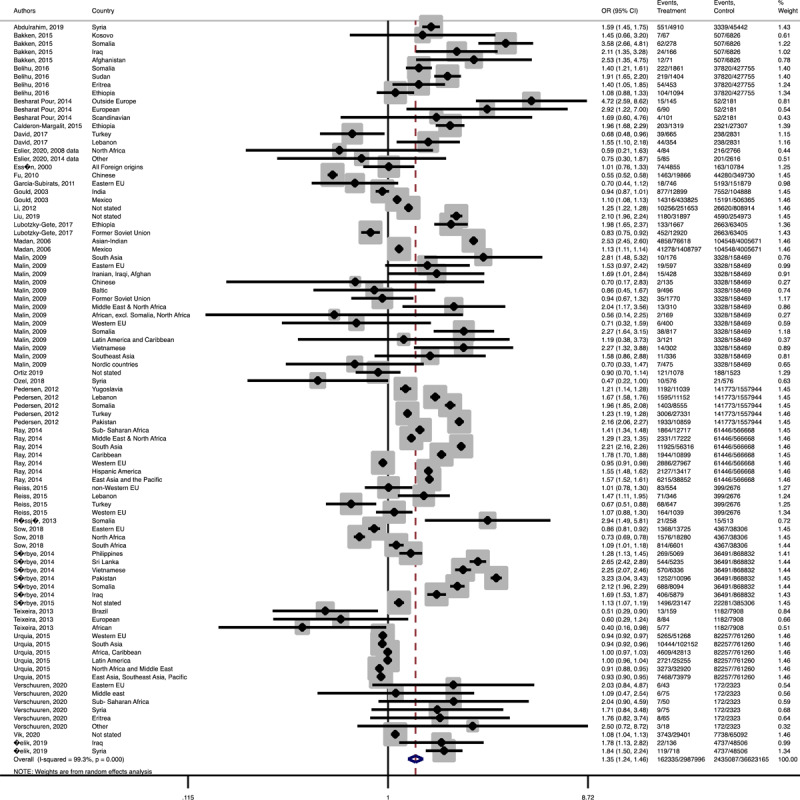
Forest plots of the pooled odds ratio of adverse neonatal outcomes in immigrant and native origin women. **(A)** Forest plot of the pooled odds ratio of small for gestational age in immigrant and native origin women.

**Figure 4-B:**
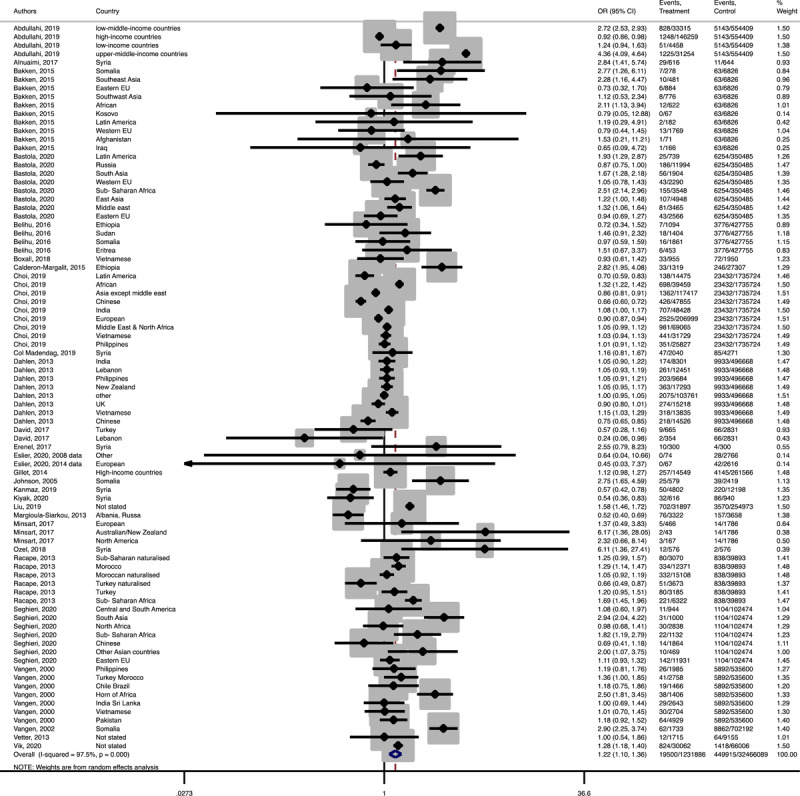
Forest plot of the pooled odds ratio of 5 min Apgar less than 7 in immigrant and native origin women.

**Figure 4-C:**
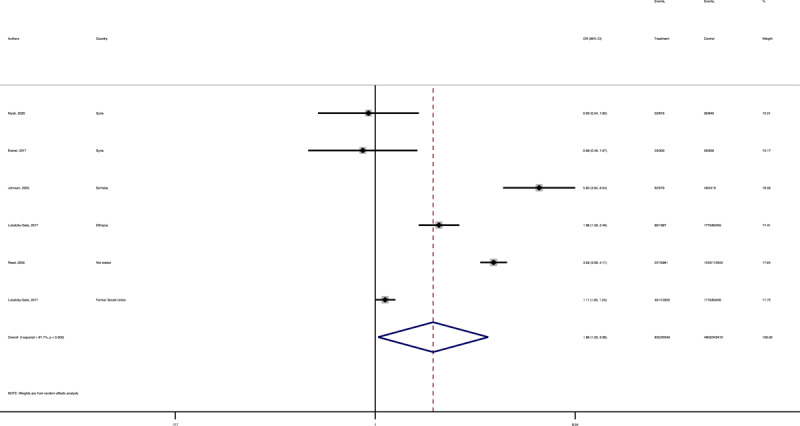
Forest plot of the pooled odds ratio of oligohydramnios in immigrant and native origin women.

**Figure 4-D:**
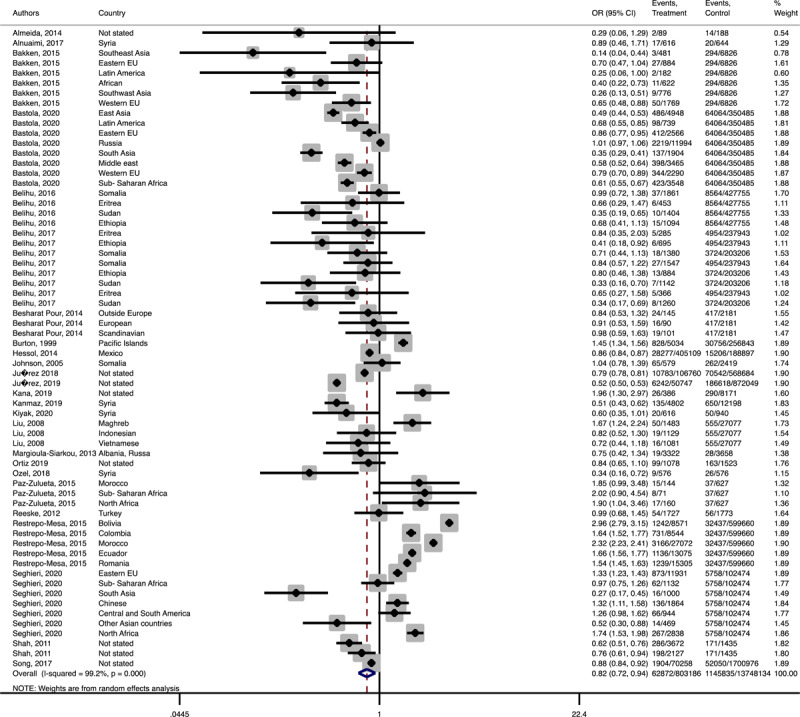
Forest plot of the pooled odds ratio of macrosomia in immigrant and native origin women.

**Figure 4-E:**
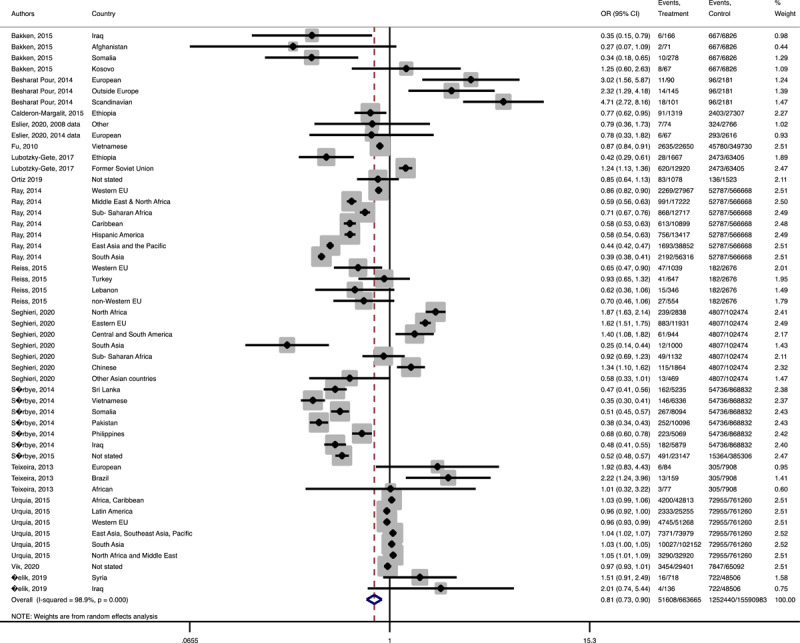
Forest plot of the pooled odds ratio of large for gestational age in immigrant and native origin women.

According to meta-regression analyses, the magnitude of those increased risks in LGA and GDM outcomes were correlated with the increased maternal age (P-value = 0.001 and 0.015, respectively) (Supplementary Table 5). However, the reported ORs were not influenced by the country of origin (Supplementary Figure 25).

## Discussion

The results of our systematic review and meta-analysis showed no consistent relationship between the immigration status and the risk of adverse maternal and neonatal events. In this respect, the risk of emergency C-S, 5-min Apgar less than 7, oligohydramnios, SGA, and GDM in the immigrant population was significantly higher in the immigrant women compared to their native origin counterparts. Nevertheless, the risk of labor induction, pregnancy-induced hypertension, preeclampsia, macrosomia, and LGA in the immigrant population was lower than the native origin population. The risks of C-S, primary C-S, instrumental delivery, preterm birth, and birth trauma were similar between the groups.

Immigration has become a social concern around the world. Conflicts, human rights violations, inequality, and job opportunities may force people to immigrate with the hope to improve their quality of life [139]. However, the increasing trends of immigration resettlement, leads to the diversity of the health status among pregnant women and new mothers in host countries. Immigrant women frequently initiate the mobility process at the childbearing age. New contexts, environments, and lifestyles may expose them to biological and psychosocial risks that tend to accentuate the situation of vulnerability [140].

In our review, heterogenous patterns in terms of the relationship between the immigration status and adverse perinatal outcomes were reported. Although available evidence is not conclusive, several hypotheses can explain the superiority or inferiority of adverse perinatal outcomes among the immigrant women compared to their counterparts in host countries. For instance, ethnic disparities in birth outcomes have been well documented [141]. Certain groups of immigrants or ethnic groups have the higher risk of suffering from adverse pregnancy outcomes. Other groups show more favorable perinatal health indicators even if they are socioeconomically vulnerable. For example, as we reported in this meta-analysis, an ethnic heterogeneity in the development of pregnancy-related hypertensive disease [142,143] or GDM [144,145,146,147,148] among western countries compared to Asian or African countries was observed. Additionally, the basic characteristics of the immigrant population may be an important sources of heterogeneity [7]. It has been reported that pregnant women with the immigration background are more likely to be younger and tend to be healthier compared to pregnant women in the native origin population [149,150]. These may suggest that the influence of immigration may be modified by those factors [151]. Our review showed that the birth outcomes such as the total and primary C-S rate, instrumental delivery, preterm birth and birth trauma are more common in the immigrant women than the native-origin women. Meanwhile, the immigrant health advantages diminish with the lower socioeconomic status including education, occupation, and family income, particularly for those immigrant women who experienced poverty in their home countries [104]. Less well-off women may get less adequate health services [152,153,154]. As well, direct associations have been reported between lifestyle and behavioral and psychological factors and adverse pregnancy outcomes [155]. The immigrant population is at the risk of particularly undocumented unhealthy life style and psychological stress, partly due to the process of cultural change [156,157], which may lead to unfavorable pregnancy outcomes. As we found in our meta-analysis, the risk of SGA as a general proxy of poverty on overall infant health/wellbeing was higher in the immigrant women compared to the native-origin women [158,159].

The risk of emergency C-S and 5-min Apgar score less than 7 in the immigrant women was higher than the native-origin population according to this review. This may be a complex interplay between the potential risk and resilience factor. Irrespective of their education level or socio-economic status in their host or country of origin, the immigrant women might have experienced poor living conditions with a limited network and language difficulties and social inequality, which might have increased the risk of life threating conditions for both the mother and child [160,161,162]. Additionally, various health and immigration policies in each host country may play a crucial role. In some countries, immigrants particularly undocumented asylum seekers face major barriers to accessing healthcare services, whereas others are more integrative and less restrictive. Differences in how immigrants are defined in each society, as well as the legal status of the immigrant population may be a challenge to access healthcare services. These may be extrapolated to prenatal care utilization, both by its timing and its content [163], which subsequently impact on the risk of adverse outcomes among pregnant women with the immigration background. Additionally, it is argued that misconceptions between healthcare providers and immigrant women through different cultural concepts and acceptability of care, or more directly through lack of interpretation services may affect pregnancy outcomes [4].

As a main strength of this study, the broad inclusion criteria and subsequent large scope of the review process at the global scale gave us an opportunity for better understanding of perinatal health among the immigrant population. However, we conclude with some final caveats and cautions. In the included studies, the different categories of immigrants groups, economic situation of immigrants, and length of residency in host countries were not specified. Therefore, subgroup analysis could not be carried out based on these classifications. In addition, the maternal duration of residence in host countries was not assessed because of lack of data. Moreover, a lack of unique definition of each adverse pregnancy outcome in the included studies may have affected this review, which should be considered during the interpretation of findings.

## Conclusion

Our meta-analysis showed heterogenous patterns with regard to the relationship between the immigrant status and adverse perinatal outcomes, as the immigrant women were at the risk of some important adverse pregnancy outcomes. Our review findings inform researchers, healthcare providers, and policymakers to pay a greater attention to the health status of pregnant women with the immigration background and also to the heterogeneity of their health outcomes in order to facilitate immigrants’ integration to the healthcare system in the host country. Population-based studies with a focus on the various aspects of adverse perinatal and neonatal outcomes in this vulnerable population are still required to improve our understanding of the sources of such heterogenicities.

## Data Availability Statement

All data come from published journal articles. Extracted data are available on a reasonable request to the corresponding author.

## Additional file

The additional file for this article can be found as follows:

10.5334/aogh.3591.s1 Supplementary Materials. Supplementary Tables 1 to 5 and Supplementary Figures 1 to 25.
