# Supplementary material for: Adverse Pregnancy Outcomes and International Immigration Status: A Systematic Review and Meta-analysis
Source: Ann Glob Health. 2022 Jun 28;88(1):44. doi: 10.5334/aogh.3591 (PMC9248985; doi:10.5334/aogh.3591)
Supplement: Supplementary Materials. — Supplementary Tables 1 to 5 and Supplementary Figures 1 to 25. [file agh-88-1-3591-s1.pdf]

Supplementary Materials

Supplementary table 1. The protocol for adverse pregnancy outcomes and international immigration status.

**Review title**	Immigration status and adverse pregnancy outcomes: A systematic review and meta-analysis
**PICO (population, intervention/Index, control, and outcomes) statement**	P: pregnant women with the history of pregnancy and their neonates; I: international immigration; C: pregnant women with the native-origin background; O: adverse maternal and neonatal outcomes.
**Anticipated or actual start date**	01.2.2019
**Anticipated completion date**	01.6.2021
**Review team**	○ Associate Professor, Samira Behboudi-Gandevani, Faculty of Health Sciences, Nord University, Bodø, Norway, Corresponding author. She conceptualizes the study and is involved in study design, search in databases, study selection, data extraction, drafting the manuscript, and revising it critically for important intellectual content.<br>○ Assistant Professor, Razieh Bidhendi-Yarandi, University of Social Welfare and Rehabilitation Sciences, Tehran, Iran, She contributes to statistical data analysis and interpreting data.<br>○ Assistant Professor Mohammad Hossein Panahi, School of Public Health and Safety, Shahid Beheshti University of Medical Sciences, Tehran, Iran. He contributes to quality assessment and interpreting data.<br>○ PhD Candidate, Abbas Mardani, Nursing Care Research Center, School of Nursing and Midwifery, Iran University of Medical Sciences. He contributes to quality and risk of bias assessment.<br>○ Professor Piret Paal, Institute of Nursing Science and Practice, Paracelsus Medical University, Austria. She contributes to interpreting data and reviewing the manuscript.<br>○ Professor Christina Prinds, University South Denmark & University College South Denmark. She contributes to interpreting data and drafting the manuscript.<br>○ Professor Mojtaba Vaismoradi, Faculty of Health Sciences, Nord University, Bodø, Norway and Faculty of Science and Health, Charles Sturt University, Australia. He is involved in search on databases, drafting the manuscript, editing, and revising it critically for important intellectual content.
**Funding sources/sponsors**	This research is performed in the framework of the research time of faculty members without any funding.
**Conflicts of interest**	None.
**Aim of study**	The objective is to investigate the risk of adverse maternal and neonatal outcomes among immigrant women compared to native-origin women in the host country.

**Identification of the research question**	○ What is the pooled risk of adverse maternal outcomes among women with the immigration background?<br>○ What is the pooled risk of adverse neonatal outcomes among women with the immigration background?
**Searches**	The following electronic bibliographic databases are searched: PubMed (including MEDLINE), Scopus, and Web of Science to retrieve relevant studies.<br><br>A manual search in the references list of selected studies and other relevant reviews will be carried out to maximize the identification of eligible studies.<br><br>A librarian at Nord University assists with the search process.
**Language**	The search strategy is restricted to English language with no time limitations.
**Keywords**	(immigration OR migration OR immigrant OR migrant OR emigrant OR asylum seeker OR asylum seeking OR asylum OR refugee) AND (“adverse pregnancy outcomes” OR “pregnancy outcomes” OR “pregnancy complications” OR abortion OR miscarriage OR “pregnancy loss” OR “fetal death” OR “stillbirth” OR “preeclampsia” OR “gestational hypertension” OR PIH OR “gestational diabetes” OR hemorrhage OR “postpartum hemorrhage” OR PPH OR “Placenta abruption” OR “placenta previa” OR preterm OR “premature rupture of membrane” OR PROM OR “Intra uterine growth restriction” OR IUGR OR “Low birth weight” OR LBW OR oligohydramnios OR Apgar OR “fetal distress” OR “neonatal distress” OR RDS OR “neonatal death” OR “neonatal mortality” OR “neonatal admission” OR “NICU admission” OR malformation OR anomalies OR “birth weight” OR LGA OR “large for gestational age” OR SGA OR “small for gestational age” OR “gestational diabetes” OR GDM OR IUFD OR “intra uterine fetal death” OR cesarean OR “operative delivery” OR “instrumental delivery” OR vacuum).
**Eligibility criteria**	Inclusion Criteria:<br><br>(i) examining immigrant women who crossed international borders;<br><br>(ii) reporting one type of data including number, prevalence, or the risk of incidents for at least one of the short-term single maternal and neonatal outcomes;<br><br>(iii) comparing those outcomes between pregnant women with the immigration background and the native-origin population<br><br>(iv) without time limitation.<br><br>Exclusion Criteria:<br><br>(i) Non-original studies including reviews, commentaries, editorials, letters, meeting abstracts, dissertations, theses, case reports, books, and conference proceedings;<br><br>(ii) lack of provision of accurate and clear data on research variables;

	(iii) duplicated data;<br><br>(v) Gray literature including governmental or organizational reports, and unpublished data.
**Condition or domain being studied**	Risk of adverse maternal and neonatal outcomes among immigrant women compared to native-origin women in the host country.
**Primary outcome(s)**	The pooled risk of developing adverse maternal outcomes among immigrant women compared to native-origin women in the host country.
**Secondary outcome(s)**	The pooled risk of developing adverse neonatal outcomes among immigrant women compared to native-origin women in the host country.
**Search strategy**	Search is performed on the mentioned databases for retrieving observational studies published up to 31. Sep. 2020, which investigates the risk of the adverse pregnancy outcomes among immigrants. Further, a manual search in the references list of selected studies and other relevant reviews is performed to maximize the search coverage.
**Data extraction (selection and coding)**	The following data are extracted: the first author's name; journal title; publication year; country; study design; sample size; population characteristics including age and body mass index; infertility definition; cancer definition, follow-up period, quality assessment and outcome measurements including the number and prevalence of cancer.<br><br>The accuracy of gathered data before the meta-analysis is assessed through double checking the data extraction process to ensure no bias in the data extraction and data entry.
**Risk of bias and quality assessment**	Two review authors independently assess the quality and risk of bias in included studies by considering the following characteristics:<br><br>Quality of the included studies is appraised using the modification of the Newcastle–Ottawa Quality Assessment Scale for Non-Randomized Studies with regard to selection, comparability and outcomes. Studies with scores above 6 are considered high quality, 4–6 moderate quality, and less than 4 low quality.<br><br>The (ROBINS) tool in non-randomized studies of interventions and observational studies is used to assess the risk of bias. Seven domains of (i) selection of exposed and non-exposed cohort, (ii) assessment of exposure, (iii) presence of outcome of interest at the start of the study, (iv) control of prognostic variables, (v) assessment of the presence or absence of prognostic factors, (vi) assessment of outcome, (vii) adequacy of follow-up are used during appraisal. The authors classify their judgment about the quality of each study into critical, moderate risk, and low risk of bias.

**Strategy for data synthesis**	A narrative synthesis of the findings is performed from the included studies, structured around the type of studies, target population characteristics, type of outcome and its content.<br><br>Meta-analysis is conducted to evaluate the pooled OR (95% CI) of the outcomes of interest using the DerSimonian and Laird and inverse variance methods.<br><br>Heterogeneity and publication bias analyses are also evaluated.<br><br>Sensitivity analysis is run to investigate the influence of each individual study on the overall meta-analysis summary estimate.<br><br>Subgroup analysis is performed based on available data about individual outcomes.<br><br>R and STATA software is used for meta-analyses.
**Equator guidelines**	The Preferred Reporting Items Systematic Reviews and Meta-analysis (PRISMA)

Supplementary table 2. Baseline characteristic of the studies' participants.

**First Author, year**	**Data sources**	**Year of data**	**Host country**	**Sample size of native origin group**	**Origin of immigrants**	**Sample size of immigrant group**
Abdullahi, 2019	Population-based data registers	1980-2010	Australia:<br>1. non-Indigenous<br>2. Indigenous	1. 509589<br>2. 44820	1. low-income countries<br>2. lower-middle-income countries<br>3. upper-middle-income countries<br>4. high-income countries	1. 4458<br>2. 33315<br>3. 31254<br>4. 146259
Abdulrahim, 2019	Population-based data registers	2011-2013	Lebanon	45442	Syria	4910
Agbemenu, 2019	Electronic birth certificate data	2007-2016	1. USA, black<br>2. USA, white	1. 5095892<br>2. 59615	African	789
Akselsson, 2020	Population-based registry	2016-2018	Sweden	26485	Somalia	623
Alder, 2008	Patient records of university hospital		Western industrialized world habited in Switzerland including<br>1. Switzerland,<br>2. Western and Northern Europe: Austria, Belgium, France, Germany, Great Britain, The Netherlands, Norway, Canada	1. 71<br>2. 21	Non-Western industrialized world habited in Switzerland including<br>1. Southern Europe: Italy, Portugal, Spain<br>2. Eastern Europe: Bosnia, Bulgaria, Yugoslav, Poland, Romania, Serbia, Kosovo, Turkey<br>3. South America: Brazil, Columbia, Dominican, Venezuela<br>4. Africa: Angola, Cameroon, Egypt, Ghana, Ivory Coast, Kenya, Tunisia<br>5. Asia: Iraq, Japan, Sri Lanka, Syria, Vietnam	1. 12<br>2. 67<br>3. 5<br>4. 9<br>5. 18
Almeida, 2014	Four public hospitals	2012	Portugal	89	Not mentioned	188
Alnuaimi, 2017	Two governmental hospitals	2014	Jordan	644	Syria	616
Badshah, 2011	Four public hospitals	2003	Pakistan	914	Afghanistan	125
Bakken, 2015	Medical birth registry of Norway and statistics of Norway	2006-2010	Norway	6826	1. Western Europe<br>2. Eastern Europe<br>3. Latin America<br>4. Southeast Asia<br>5. Southwast Asia<br>6. Africa	1. 1769<br>2. 884<br>3. 182<br>4. 481<br>5. 776<br>6. 622
Bakken, 2015	Medical birth registry of Norway and statistics of Norway	2006-2010	Norway	6826	1.Somalia<br>2. Iraq<br>3. Afghanistan<br>4. Kosovo	1. 278<br>2. 166<br>3. 71<br>4. 67
Bakken, 2017	One hospital	2006-2013	Norway	8237	1. Pakistan, first generation<br>2. Pakistan, second generation	1. 211<br>2. 76
Bastola, 2020	Medical birth register of<br>Finland	2004-2014	Finland	350485	1. Western EU<br>2. Eastern EU<br>3. Russia<br>4. South Asia<br>5. East Asia<br>6. Sub‐Saharan Africa<br>7. Middle East<br>8. Latin America	1. 2290<br>2. 2566<br>3. 11994<br>4. 1904<br>5. 4948<br>6. 3548<br>7. 3465<br>8. 739
Bastola, 2019	Medical birth register and the hospital discharge register	2004-2014	Finland	243	1. Russia<br>2. Somali<br>3. Kurdish	1. 348<br>2. 584<br>3. 373
Bastola, 2019	Medical birth register and the hospital discharge register	2004-2014	Finland	243	1. Russia<br>2. Somali<br>3. Kurdish	1. 318<br>2. 584<br>3. 373
Belihu, 2016	Victorian routine perinatal data registry	1999-2007	Australia	427755	1. Eritrea<br>2. Ethiopia<br>3. Somalia<br>4. Sudan	1. 453<br>2. 1094<br>3. 1861<br>4. 1404
Belihu, 2017	Victorian perinatal data collection	1999-2007	Australia	203206	1. Eritrea<br>2. Ethiopia<br>3. Somalia<br>4. Sudan	1. 285<br>2. 695<br>3. 1380<br>4. 1142
Belihu, 2017	Victorian perinatal data collection	1999-2007	Australia	237943	1. Eritrea<br>2. Ethiopia<br>3. Somalia<br>4. Sudan	1. 366<br>2. 884<br>3. 1547<br>4. 1260
Bernis, 2013	One university public hospital	2005-2007	Spain	10965	1. Eastern Europe<br>2. Central America	1. 903<br>2. 493

					3. South America<br>4. Maghreb<br>5. China	3. 3208<br>4. 782<br>5. 247
Biro, 2017	Three hospitals	2013-2015	Australia	18020	Afghanistan, Benin, Bhutan, Burma, Burundi, Chad, Congo, Guinea, Iraq, Ivory Coast, Liberia, Rwanda, Sierra Leone, Stateless, Sudan, Tibet, Togo, Uganda	1547
Boxall, 2018	One hospital	2010-2015	Germany	1950	Vietnam	955
Bozorgmehr, 2018	Referral hospital	2010-2016	Germany	19259	Not Mentioned	569
Breckenkamp, 2019	Three hospitals	2011-2012	Germany	1208	1. Turkey<br>2. Lebanon	1. 133<br>2. 72
Burton, 1999	New South Wales midwives data collection	1990-1993	Australia	256843	Pacific Islands	5034
Calderon-Margalit, 2015	One medical center	2002-2009	Israel	27307	Ethiopia	1319
Castelló, 2012	Hospital records registry	1997-2008	Spain	15782	1. Eastern Europe: Rumania, Russia, Lithuania, Bulgaria, Ukraine<br>2. Maghreb<br>3. Sub-Sahara: Senegal, Guinea Bissau, Nigeria, Ghana, Malawi<br>4. Latin America: Ecuador, Bolivia, Colombia, Peru, Argentina, Brazil	1. 2149<br>2. 2648<br>3. 608<br>4. 521
Çelik, 2019	One hospital	2013-2016	Turkey	48506	1. Syria<br>2. Iraq	1. 718<br>2. 136
Choi, 2019	Australian national perinatal data collection	2004 - 2013	Australia	1735724	1. Europe<br>2. Middle East and North Africa<br>3. China<br>4. India<br>5. Philippines<br>6. Vietnam<br>7. Asia except middle east<br>8. Latin America<br>9. Africa	1. 206999<br>2. 69065<br>3. 47855<br>4. 48428<br>5. 25827<br>6. 31729<br>7. 117417<br>8. 14475<br>9. 39459
Col Madendag, 2019	One hospital database	2018-2019	Turkey	4271	Syria	2040
Dahlen, 2013	New South Wales midwives data collection	2000-2008	Australia	496668	1. New Zealand<br>2. England<br>3. China<br>4. Vietnam<br>5. Lebanon<br>6. Philippines<br>7. India<br>8. other	1. 17293<br>2. 15218<br>3. 14526<br>4. 13835<br>5. 12451<br>6. 9684<br>7. 8301<br>8. 103761
David, 2017	Three hospitals	2011-2012	Germany	2831	1. Turkey<br>2. Lebanon	1. 665<br>2. 354
Dejin-Karlsson, 2004	Population registry, pregnancy outcome database of university hospital	1991-1992	Sweden	644	1. Eastern Europe<br>2. Middle East and North Africa<br>3. Central and South America<br>4. Asia<br>5. Sub-Saharan Africa	1. 50<br>2. 49<br>3. 14<br>4. 24<br>5. 13
Demirci, 2017	One hospital	2015	Turkey	545	Syria	545
Erenel, 2017	One hospital	2013-2016	Turkey	300	Syria	300
Eskild, 2020	Medical birth registry of Norway, central person registry of Norway	1999-2014	Norway	668439	1. Somalia<br>2. Afghanistan<br>3. Iraq	1. 9281<br>2. 2113<br>3. 7423
Eslier, 2020	One hospital	2008 and 2014	France	2008: 2766<br>2014: 2616	1. Europe<br>2. North Africa<br>3. Sub-Saharan Africa<br>4. Other	2008:<br>1. 40<br>2. 84<br>3. 74<br>4. 74<br>2014:<br>1. 67<br>2. 105<br>3. 128<br>4. 85
Essén, 2000	Malmö database, Swedish medical birth register	1990-1995	Sweden	4855	All Foreign origins	356

Fu, 2010 101	Taiwan’s nation-wide birth certificate data	2006-2007	Taiwan	349730	1. China<br>2. Vietnam	1. 19866<br>2. 22650
Gagnon, 2013	NORMAPERS study database	2003-2004	Canada	51	1. Europe<br>2. Latin America<br>3. Africa<br>4. North-Africa and West- Asia<br>5. South-East and Central Asia	1. 19<br>2. 30<br>3. 28<br>4. 8<br>5. 55
Garcia-Subirats, 2011	Births registry	1991-2005	Spain	145868	1. Latin America<br>2. Asia<br>3. Eastern Europe<br>4. Maghrib countries	1. 8722<br>2. 2445<br>3. 734<br>4. 1906
Gillet, 2014	Study center for perinatal epidemiology, Belgian civil birth registration system	2204-2008	Belgium	261566	Low-income countries<br>Middle-income countries<br>High-income countries	8066<br>41985<br>14549
Gould, 2003	California linked infant birth/death certificate files	1995-1997	1. USA, White<br>2. USA, Black	1. 506365<br>2. 104888	1. India<br>2. Mexico	1. 12899<br>2. 433825
Güngör, 2018	One hospital	2016-2017	Turkey	744	Syria	704
Hamilcikan, 2017	One hospital	2015-2016	Turkey	2083	Not mentioned	1026
Harding, 2006	Hospital records	2001-2002	Portugal	2949	African	461
Hessol, 2014	Birth certificate data	2003	USA	188897	Mexico	405109
Hsieh, 2011	Birth certificate registration data	1998-2003	Taiwan	1321770	Not mentioned	93161
Johnson, 2005	Birth certificate data	1993-2001	1. USA, black<br>2. USA, white	1. 2384<br>2. 2453	Somali	579
Juárez, 2019	Longitudinal database for integration studies, Swedish medical birth, multi-generational register	1997-2012	Sweden	872049	Not mentioned	77544
Juárez, 2014	National institute of statistics	2009–2011	Spain	1061924	1. Western EU<br>2. Eastern EU<br>3. Non-EU Europe<br>4. North Africa<br>5. Sub-Saharan Africa<br>6. USA and Canada<br>7. Central America & Mexico<br>8. Caribbean<br>9. South America<br>10. Asia	1. 25798<br>2. 48025<br>3. 9010<br>4. 83466<br>5. 14217<br>6. 1279<br>7. 4390<br>8. 12308<br>9. 103445<br>10. 21918
Juárez 2017	Swedish medical birth register	1999-2012	Sweden	568684	1. Finland<br>2. Norway<br>3. Denmark<br>4. Germany<br>5. Poland<br>6. Syria<br>7. Iraq<br>8. Iran<br>9. Lebanon<br>10. Turkey<br>11. F. Yugoslavia<br>12. Ethiopia<br>13. Somalia<br>14. India<br>15. South Korea<br>16. Thailand<br>17. Chile<br>18. Rest	1. 7552<br>2. 3167<br>3. 2676<br>4. 2789<br>5. 5292<br>6. 5528<br>7. 20770<br>8. 748<br>9. 6898<br>10. 7488<br>11. 18778<br>12. 2899<br>13. 11235<br>14. 2048<br>15. 2081<br>16. 5057<br>17. 3860<br>18. 48862
Juárez 2018	Swedish medical birth register	1999-2012	Sweden	473881	1. Finland<br>2. Norway<br>3. Denmark<br>4. Germany<br>5. Poland<br>6. Syria<br>7. Iraq<br>8. Iran<br>9. Lebanon<br>10. Turkey<br>11. F. Yugoslavia	1. 4379<br>2. 2079<br>3. 1797<br>4. 2173<br>5. 4790<br>6. 2662<br>7. 12201<br>8. 4766<br>9. 2800<br>10. 4055<br>11. 13536

					12. Ethiopia<br>13. Somalia<br>14. India<br>15. South Korea<br>16. Thailand<br>17. Chile<br>18. Rest	12. 1338<br>13. 3030<br>14. 2171<br>15. 1918<br>16. 3589<br>17. 2398<br>18. 37078
Kana, 2019 1	Generation XXI birth cohort	2005-2006	Portuguese	8171	Not mentioned	386
Kandasamy, 2014	One hospital	2008-2010	Canada	273	1. Latin America and the Caribbean<br>2. Western Europe/USA/Japan/ Australia<br>3. Eastern Europe and Central Asia<br>4. Middle East and North Africa<br>5. Sub-Saharan Africa<br>6. South Asia<br>7. East Asia and the Pacific	1. 148<br>2. 4<br>3. 16<br>4. 1<br>5. 64<br>6. 5<br>7. 34
Kane, 2018	Electronic birth certificate records	1999-2012	USA	1387173	India	65375
Kanmaz, 2019	One hospital	2013-2016	Turkey	12198	Syria	4802
Khanolkar, 2015	Medical birth registry	1982-2002	Sweden	1435286	1. Western Europe and North America<br>2. Finland<br>3. Eastern Europe<br>4. Poland<br>5. Yugoslavia<br>6. North Africa and Middle East<br>7. Iraq<br>8. Lebanon<br>9. Somalia<br>10. Syria<br>11. Turkey<br>12. Iran<br>13. South Asia<br>14. Ethiopia and Eritrea<br>15. East Asia<br>16. Latin America<br>17. Chile<br>18. Sub-Saharan Africa	1. 4798<br>2. 12124<br>3. 3112<br>4. 3510<br>5. 16986<br>6. 3985<br>7. 9245<br>8. 6960<br>9. 3593<br>10. 3963<br>11. 11462<br>12. 7898<br>13. 4300<br>14. 4364<br>15. 5817<br>16. 1940<br>17. 4186<br>18. 2112
Kiyak, 2020	One hospital	2016-2017	Turkey	940	Syria	616
Krishnakumar, 2011	Hospitals record	1996-2003	1. USA white<br>2. USA black	1. 129975<br>2. 10667	1. Immigrant White<br>2. Immigrant Black	1. 5205<br>2. 764
Li, 2013	National research database, the womMed II database, medical birth register	1982-2006	Sweden	1874567	Denmark, Finland, Norway, Greece, Italy, Great Britain/Ireland, Germany, Austria, Yugoslavia, Croatia, Romania, Bulgaria, Estonia, Poland, Hungary, Chile, Turkey, Lebanon, Iran, Iraq, Russia	318276
Li, 2012	National research database, the womMed II database, medical birth register	1982-2006	Sweden	808914	Denmark, Finland, Norway, France, Greece, Italy, Spain, The Netherlands, Great Britain/Ireland, Germany, Austria, Yugoslavia, Croatia, Romania, Bulgaria, Estonia, Poland, Hungary, Russia, Chile, Turkey, Lebanon, Iran, Iraq	251653
Liu, 2019	Swedish pregnancy register	2014-2017	Sweden	254973	Not mentioned	31897
Liu, 2008	Birth certificates	2002-2007	Taiwan	27077	1. Mainland Chinese<br>2. Indonesian<br>3. Vietnamese	1. 1483<br>2. 1129<br>3. 1081
Lubotzky-Gete, 2017	One hospital	1998-2011	Israel	63405	Ethiopia<br>Former Soviet Union	1. 1667<br>2. 12920
Ma, 1996	New South Wales midwives data collection	1990-1992	Australia	189357	1. Europe<br>2. Asia<br>3. Middle east<br>4. America<br>5. Newzland<br>6. Africa	1. 20049<br>2. 19458<br>3. 10171<br>4. 3363<br>5. 9522<br>6. 2359
Madan, 2006	Birth certificates, perinatal mortality data file	1995-2000	US-born white	4005671	1. Asia-India<br>2. Mexico	1. 76618<br>2. 1408797
Malamitsi-Puchner, 1994	One hospital	1990-1991	Greece	1231	1. Eastern Europe<br>2. Middle East<br>3. Africa<br>4. Pontian	1. 247<br>2. 179<br>3. 68<br>4. 144
Malin, 2009	Finnish medical birth	1999-	Finland	158469	1. Nordic	1. 475

	register, statistics Finland	2001			2. Western EU<br>3. Eastern Europe<br>4. Former Soviet Union, Russia<br>5. Baltic<br>6. Middle East, North-Africa<br>7. South Asia<br>8. China<br>9. Iran, Iraq, Afghanistan<br>10. Southeast Asia<br>11. Vietnam<br>12. African, excl. Somalia, North Africa<br>13. Somali<br>14. Latin America and Caribbean	2. 400<br>3. 597<br>4. 1770<br>5. 496<br>6. 310<br>7. 176<br>8. 135<br>9. 428<br>10. 336<br>11. 302<br>12. 169<br>13. 817<br>14. 121
Margioula-Siarkou, 2013	One hospital record	2003-2009	Greece	3658	Albania, Russia	3322
Minsart, 2017	One hospital	2012-2015	China	1786	1. North America<br>2. Europe<br>3. Australi/New Zealand	1. 167<br>2. 466<br>3. 43
Moore, 2009	Que´bec birth registry	2002	Canada	42174	1. North America/western Europe<br>2. Eastern Europe<br>3. Sub-Saharan Africa<br>4. Middle East and North Africa<br>5. East Asia and the Pacific<br>6. South Asia<br>7. Latin America<br>8. Caribbean	1. 1025<br>2. 361<br>3. 347<br>4. 1182<br>5. 752<br>6. 542<br>7. 519<br>8. 1086
Mozooni, 2020	Perinatal, birth, death, hospital, birth defects registrations	2005-2013	Australia	172571	1. White<br>2. Asia<br>3. India<br>4. Africa<br>5. Māori<br>6. Other	1. 48546<br>2. 18212<br>3. 5503<br>4. 4155<br>5. 2941<br>6. 9038
Naimy, 2013	Medical birth registry of Norway, Norwegian central person registry	1986-2005	Norway	1062744	1. Pakistan<br>2. Vietnam<br>3. Somalia<br>4. Sri Lanka<br>5. Philippines<br>5. Iraq<br>6. Thailand<br>7. Afghanistan	1. 11351<br>2. 6169<br>3. 5410<br>4. 4933<br>5. 4662<br>6. 3829<br>7. 3204<br>8. 665
Naimy, 2015	Medical birth registry of Norway, Norwegian central person registry	1986-2005	Norway	1062744	1. Pakistan<br>2. Vietnam<br>3. Somalia<br>4.Sri Lanka<br>5. Philippines<br>6. Iraq<br>7. Thailand<br>8. Afghanistan	1. 11351<br>2. 6169<br>3. 5410<br>4. 4933<br>5. 4662<br>6. 3829<br>7. 3204<br>8. 665
Kragelund Nielsen, 2020	Danish medical birth registry	2004-2015	Denmark	621154	1. Sweden<br>2. Norway<br>3. Former Soviet<br>4. Germany<br>5. Iceland<br>6. USA<br>7. Other Western countries<br>8. Great Britain<br>9. Poland<br>10. Romania<br>11. Yugoslavia<br>12. Syria<br>13. Lebanon<br>14. Vietnam<br>15. Thailand<br>16. Other Non-western countries<br>17. Somalia<br>18. Philippines<br>19. Afghanistan<br>20. Iran<br>21. Turkey<br>22. Iraq<br>23. China<br>24. Pakistan<br>25. India<br>26. Morocco<br>27. Sri Lanka	1. 2738<br>2. 3000<br>3. 7777<br>4. 3253<br>5. 2261<br>6. 1014<br>7. 6332<br>8. 1146<br>9. 5240<br>10. 2234<br>11. 7211<br>12. 1768<br>13. 4530<br>14. 2633<br>15. 2739<br>16. 12111<br>17- 5539<br>18. 2362<br>19. 3281<br>20. 1996<br>21. 8183<br>22. 6150<br>23. 2218<br>24. 3909<br>25. 1132<br>26. 1898<br>27.1673

Nilsen, 2018	Medical birth registry of Norway, statistics Norway	1990-2013	Norway	1123762	Nordic countries	22594
Opondo, 2020	Statutory birth and death registration data for England and Wales, National Health Service Numbers for Babies (NN4B) birth notifications system, office for national statistics	2006-2012	UK	3009231	1. White (other)<br>2. India<br>3. Pakistan<br>4. Bangladesh<br>5. Black Caribbean<br>6. Black Africa<br>7. Mixed or other<br>8. Not stated	1. 340526<br>2. 132651<br>3. 180651<br>4. 62948<br>5. 47505<br>6. 154076<br>7. 419970<br>8. 287756
Ortiz 2019	One hospital	2015	Chile	1523	Peru, Colombia, Haiti, Bolivia, Dominican, Ecuador, Argentine, Venezuela, China, Brazil, Cuba, Spain, Uruguay, Others	1078
Ozel, 2018	One hospital	2015	Turkey	576	Syria	576
Park, 2015	Live birth records provided by vital statistics	2002-2011	Canada	670492	1. South Korea<br>2. Eritrea<br>3. Bosnia<br>4. Lybia<br>5. Somalia<br>6. China<br>7. Brazil<br>8. Lebanon<br>9. Colombia<br>10. Srbia and Montenegro<br>11. Saudi Arabia<br>12. Afghanestan<br>13. Russia<br>14. Ethiopia<br>15. Ukraine<br>16. Kuwait<br>17. USA<br>18. Sudan<br>19. Mexico<br>20. Israel<br>21. Iran<br>22. Argentina<br>23. Poland<br>24. Irag<br>25. Syria<br>26. Hong Kong<br>27. UK<br>28. Romania<br>29. Vietnam<br>30. Turkey<br>31. Egypt<br>32. Portugal<br>33. Albania<br>34. South Africa<br>35. Nigeria<br>36. Pakistan<br>37. India<br>38. Srilanka<br>39. Bulgaria<br>40. Elsalvador<br>41. Bangladesh<br>42. Hungary<br>43. Ecuador<br>44. Haiti<br>45. Philippine<br>46. Ghana<br>47. Jamaica<br>48. Trinidad and Tobago<br>49. Congo<br>50. Guyana	1. 3451<br>2. 789<br>3. 1248<br>4. 886<br>5. 4833<br>6. 31976<br>7. 1090<br>8. 4638<br>9. 2179<br>10. 1560<br>11. 897<br>12. 6392<br>13. 2014<br>14. 2726<br>15. 1943<br>16. 833<br>17. 1694<br>18. 1638<br>19. 7002<br>20. 1005<br>21. 5065<br>22. 722<br>23. 5981<br>24. 5406<br>25. 1159<br>26. 2779<br>27. 1951<br>28. 4287<br>29. 10039<br>30. 2100<br>31. 2679<br>32. 3120<br>33. 1428<br>34. 692<br>35. 3107<br>36. 26683<br>37. 46799<br>38. 20243<br>39. 761<br>40. 1449<br>41. 5481<br>42. 746<br>43. 744<br>44. 920<br>45. 17017<br>46. 2729<br>47. 8688<br>48. 1965<br>49. 816<br>50. 5590
Paz-Zulueta, 2015	Clinical databases of primary health care centers	2007-2010	Spain	627	1. North African<br>2. Moroco<br>3. Sub-Saharan<br>4. Spain<br>5. North African<br>6. Morocco<br>7. Sub-Saharan<br>8. Spain	1. 160<br>2. 144<br>3. 71<br>4. 627<br>5. 160<br>6. 144<br>7. 71<br>8. 627

					9. North African<br>10. Morocco<br>11. Sub-Saharan	9. 160<br>10. 144<br>11. 71
Pedersen, 2012	Danish medical birth registry	1978-2007	Denmark	1557944	1. Turkey<br>2. Yugoslavia<br>3. Lebanon<br>4. Pakistan<br>5. Somali<br>6. Denmark<br>7. Turkey<br>8. Yugoslavia<br>9. Lebanon<br>10. Pakistan<br>11. Somali	1. 27331<br>2. 11039<br>3. 11152<br>4. 10859<br>5. 8555<br>6. 1557944<br>7. 27331<br>8. 11039<br>9. 11152<br>10. 10859<br>11. 8555
Besharat Pour, 2014	Swedish prospective birth cohort study of barn/children allergy milieu Stockholm epidemiology	1994-1996	Sweden	2181	1. Scandinavia<br>2. Europe<br>3. Outside Europe	1. 101<br>2. 90<br>3. 145
Racape, 2013	Birth and death certificates	1998-2008	Belgium	39893	1. Morocco<br>2. Moroccan naturalised<br>3. Sub-Sahara<br>4. Sub-Saharan naturalised<br>5. Turkey<br>6. Turkey naturalised	1. 12371<br>2. 15108<br>3. 6322<br>4. 3070<br>5. 3185<br>6. 3673
Racape, 2016	Birth and death certificates	1998-2010	Belgium	1029471	1. Western Euroupe<br>2. Western Euroupe naturalized Belgian<br>3. Turkey<br>4. Turkey naturalized Belgian<br>5. Maghreb<br>6. Maghreb naturalized Belgian<br>7. Sub-Saharan Africa<br>8. Sub-Saharan Africa naturalized Belgian<br>9. Eastern Europe<br>10. Eastern Europe naturalized Belgian	1. 98189<br>2. 34701<br>3. 20451<br>4. 21878<br>5. 51224<br>6. 46681<br>7. 26621<br>8. 11420<br>9. 17420<br>10. 5412
Raimondi, 2013	Public maternity hospital	2009	Argentine	1000	Not mentioned	1000
Ray, 2014	Datasets at the institute for clinical evaluative sciences	2002-2008	Canada	566668	1. Sub-Sahara<br>2. South Asia<br>3. Caribbean<br>4. Hispanic America<br>5. Middle East & North Africa<br>6. Western Europe<br>7. East Asia and Pacific	1. 12717<br>2. 56316<br>3. 10899<br>4. 13417<br>5. 17222<br>6. 27967<br>7. 38852
Reed, 2005	Medicaid data, birth certificate data	1998-1999	USA	112943	Not mentioned	5961
Reeske, 2012	Pregnancy-related health insurance data	2005-2007	Germany	1691	Turkey	1647
Reiss, 2015	Three hospitals	2011-2012	Germany	2676	Turkey<br>Lebanon<br>non-Western EU<br>Western EU	1. 647<br>2. 346<br>3. 554<br>4. 1039
Restrepo-Mesa, 2015	Birth registry	2007-2008	Spain	599660	1. Romania<br>2. Morocco<br>3. Bolivia<br>4. Colombia<br>5. Ecuador	1. 15305<br>2. 27072<br>3. 8571<br>4. 8544<br>13075
Råssjö, 2013	Records of antenatal and obstetric care	2001-2009	Sweden	513	Somalia	258
Sanchalika, 2015	Birth certificate data, hospitalization data	1999-2002	USA, white	308508	1. India<br>2. Bangladesh<br>3. Pakistan<br>4. Sri-Lanka	1. 14612<br>2. 833<br>3. 2924<br>4. 192
Scholaske, 2019	German socio-economic panel data	2002-2016	Germany	2308	Turkey	217
Sdona, 2019	Public maternity hospital	2010-2014	Greece	5154	Not mentioned	1971
Seghieri, 2020	Database of certificates of care at delivery	2012–2017	Italy	102474	1. North Africa<br>2. Sub-Sahara<br>3. South Asia<br>4. China<br>5. Other Asian countries<br>6. Central/South America<br>7. Eastern Europe	1. 2838<br>2. 1132<br>3. 1000<br>4. 1864<br>5. 469<br>6. 944<br>7. 11931
Shah, 2011	Hospital	2002-	Canada	1435	Not mentioned	3672

		2006				2127
Sole, 2018	Medical birth registry of Norway and statistics Norway	1999-2014	Norway	724102	1. Western Europe<br>2. Eastern Europe<br>3. North America<br>4. Latin America/Caribbean<br>5. Middle East/North Africae<br>6. Sub Saharan Africa<br>7. Transcaucasia/Central Asia<br>8. South Asia<br>9. East Asia Pacific<br>10. Oceania, Australia, NZ	1. 54859<br>2. 21533<br>3. 3983<br>4. 7847<br>5. 19552<br>6. 24029<br>7. 806<br>8. 19388<br>9. 25457<br>10. 535
Song, 2017 250	Korean national birth registry	2010-2013	Korea	1700976	Not mentioned	70258
Sørbye, 2015	Birth registry data, immigration data	1990-2009	Norway	385306	1. Pakistan<br>2. Vietnam<br>3. Philippines<br>4. Poland<br>5. Sri Lanka<br>6. Yugoslav countries<br>7. Iraq<br>8. Somalia<br>9. Thailand<br>10. Turkey	1. 3086<br>2. 2695<br>3. 2457<br>4. 2400<br>5. 2265<br>6. 2187<br>7. 2165<br>8. 2014<br>9. 1965<br>10. 1913
Sørbye, 2014	Statistic Norway, medical birth registry of Norway	1990–2009	Norway	868832	1. Pakistan<br>2. Somalia<br>3. Vietnam<br>4. Iraq<br>5. Sri Lanka<br>6. Philippines	1. 10096<br>2. 8094<br>3. 6336<br>4. 5879<br>5. 5235<br>6. 5069
Sosta, 2008	University hospital	2005	Italy	366	1. Africa<br>2. East Europe<br>3. Asia<br>4. South America	1. 29<br>2. 44<br>3. 21<br>4. 11
Sow, 2018	Birth and death statistical reports for Brussels residents, the national registry, “Banque carrefour de la sécurité sociale” (BCSS, Crossroads Bank for Social Security)	2005-2010	Belgium	39155	1. East Europe<br>2. North Africa<br>3. South Africa<br>4. Belgium<br>5. East Europe<br>6. North Africa<br>7. South Africa	1. 14060<br>2. 18616<br>3. 6742<br>4. 38670<br>5. 13830<br>6. 18428<br>7. 6657
Sow, 2019	Brussels birth and death registers, the national register of migrant trajectories, social security register	2004-2010	Belgium	27265	1. Maghreb<br>2. Sub-Saharan<br>3. Turkey	1. 32304<br>2. 9778<br>3. 6175
Sunil, 2012	Migration of Mexican households,<br>USA infant health outcomes, Encuesta Nacional de la Dinamica Demografica	2006	USA	38000	Mexico	2649
Teixeira, 2013	Public level III maternities	2005-2006	Portuguese	7908	1. European<br>2. African<br>3. Brazilian	1. 84<br>2. 77<br>3. 159
Tsimbos, 2011	Vital registration system	2006	Greece	85676	Not mentioned	17590
Turkay, 2020	One hospital	2016-2017	Turkey	7950	Syria	620
Urquia, 2015	Institute for clinical evaluative sciences database	2002-2012	Canada	761260	1. Western Europe<br>2. Africa, Caribbean<br>3. North Africa, Middle East<br>4. Latin America<br>5. East Asia, Southeast Asia, Pacific<br>6. South Asia	1. 51268<br>2. 42813<br>3. 32920<br>4. 25255<br>5. 73979<br>6. 102152
Urquia, 2010	Hospital records, discharge abstract database of the Canadian institute for health information	2002-2007	Canada	314237	Central and Eastern Europe, Middle East and North Africa, Sub-Saharan Africa, Caribbean Hispanic America, East Asia & Pacific, South Asia	83233
Urquia, 2015, Canada data	National birth certificate data	2000–2005	Canada	1427351	1. Argentina<br>2. Bolivia<br>3. Chile	1. 1623<br>2. 608<br>3. 1986

					4. Colombia<br>5. Cuba<br>6. Ecuador<br>7. El Salvador<br>8. Honduras<br>9. Mexico<br>10. Nicaragua<br>11. Paraguay<br>12. Peru<br>13. Uruguay<br>14. Venezuela	4. 2731<br>5. 911<br>6. 1303<br>7. 4237<br>8. 565<br>9. 9780<br>10. 1049<br>11. 615<br>12. 1869<br>13. 459<br>14. 1035
Vahratian, 2004	Three hospitals	1994-1995	Belgium	808	North Africa	354
Vangen, 2002	Medical birth registry of Norway	1986-1998	Norway	702192	Somalia	1733
Vangen, 2000	Medical birth registry of Norway	1986-19958	Norway	535600	1. Turkey, Morocco<br>2. Pakistan<br>3. India Sri Lanka<br>4. Vietnam<br>5. Philippines<br>6. Horn of Africa<br>7. Chile Brazil	1. 2758<br>2. 4929<br>3. 2643<br>4. 2704<br>5. 1985<br>6. 1406<br>7. 1466
Verschuuren, 2020	One midwifery practice, hospital databases	2012-2016	Netherlands	2323	1. Eritrea<br>2. Syria<br>3. Middle east<br>4. Sub-Saharan Africa<br>5. Eastern Europe<br>6. Other	1. 65<br>2. 75<br>3. 75<br>4. 50<br>5. 43<br>6. 18
Vetter, 2013	12 public hospitals	2008	Argentine	9155	Not mentioned	1715
Vik, 2019	Medical birth registry of Norway and statistics Norway	1990-2013	Norway	1136637	Central Europe, Eastern Europe, Central Asia, High-income, Latin America, Caribbean, North Africa, Middle East, South Asia, Sub-Saharan Africa, Southeast Asia, East Asia, Oceania, Other	195725
Vik, 2020	Medical birth registry of Norway and statistics Norway	1990–2016	Norway	66006	Not mentioned	30062
Villadsen, 2010	Birth registries or surveys	1990–2005	1. Belgium<br>2. Denmark<br>3. Norway<br>4. Sweden<br>5. Switzerland<br>6. Austria<br>7. UK<br>8. Germany<br>9. Netherlands	1. 238233<br>2. 812305<br>3. 675387<br>4. 1344237<br>5. 900875<br>6. 408695<br>7. 1037348<br>8. 1296798<br>9. 935858	Turkey	8717
Walsh, 2011	Tertiary referral centre	2008	Ireland	2045	Eastern European	511
Wanigaratne, 2016	Immigration records, Ontario hospital data	2002-2011	Canada	860617	1. Refugees<br>2. Other immigrants	1. 29765<br>2. 230914
Wanigaratne, 2018	IRCC-PRD, Ontario's healthcare registry, abstract database, general's vital statistics- death registry	2002-2014	Canada	29023	Not mentioned	29023
Xirasagar, 2011	One hospital	2002-2007	Taiwan	2646	1. China<br>2. Vietnam	1. 341<br>2. 280
Yoong, 2004	One teaching hospital	2002	UK	61	Kosovo, Albanians	61
Zanconato, 2011	One public hospital	2005-2009	Italy	6365	1. Central and Eastern Europe<br>2. South and East Asia<br>3. Middle East and North Africa<br>4. Sub-Saharan Africa<br>5. Central and South America	1. 1001<br>2. 539<br>3. 460<br>4. 448<br>5. 213
Zeitlin, 2011	Medical records	1999-2001	France	21727	1. North Africa<br>2. Sub-Saharan Africa<br>3. Other countries	1. 9325<br>2. 7187<br>3. 9828
Zuppa, 2010	One hospital	2005	Italy	2334	Not mentioned	585

Supplementary table 3. Quality assessment of studies included using the Newcastle–Ottawa Quality Assessment Scale for cohort studies.

	**SELECTION**				**COMPARABILITY**	**OUTCOME**			**Total scores**	
**Author, Year**	Representativeness of the exposed cohort *	Selection of the non-exposed cohort *	Ascertainment of exposure *	No outcome of interest at the start of the study *	A: Study controls for age and/or BMI *<br>B: Study controls for *other confounders	A: doctor's diagnosis OR objective measurements<br>B: parent/self-reported doctor's diagnosis OR use of medication	Follow-up long enough for outcomes *	Adequacy of follow up of cohorts *		
Abdullahi et al, 2019	*	*	*	*	**	*	*	*	9*	High
Alder et al, 2008			*	*	**	*	*	*	7*	High
Alnuaimi et al, 2017			*	*		*	*	*	5*	Moderate
Badshah et al, 2011			*	*	**	*	*	*	7*	High
Bakken, 2015	*	*	*	*	**	*	*	*	9*	High
Biro and East, 2017			*	*	**	*	*	*	7*	High
Boxall et al, 2018			*	*	**	*	*	*	7*	High
Burton, 1999	*	*	*	*	**	*	*	*	9*	High
ÇELIK et al, 2019			*	*		*	*	*	5*	Moderate
Choi et al, 2019	*	*	*	*	**	*	*	*	9*	High
Col Madendag et al, 2019			*	*	**	*	*	*	7*	High
Dejin-Karlsson et al, 2004	*	*	*	*	**	*	*	*	9*	High
Demirci et al, 2017			*	*		*	*	*	5*	Moderate
Eslier et al, 2020			*	*	*	*	*	*	6*	Moderate
Essen et al, 2000	*	*	*	*	**	*	*	*	9*	High
Güngör et al, 2018			*	*		*	*	*	5*	Moderate
Johnson et al, 2005	*	*	*	*	**	*	*	*	9*	High
Kandasamy et al, 2014			*	*		*	*	*	5*	Moderate
Kanmaz et al, 2019			*	*	**	*	*	*	7*	High
Kiyak et al, 2020			*	*		*	*	*	5*	Moderate
Li et al, 2013	*	*	*	*	**	*	*	*	9*	High
Li et al, 2012	*	*	*	*	**	*	*	*	9*	High
Liu et al, 2019	*	*	*	*	**	*	*	*	9*	High
Lubotzky-Gete et al, 2017			*	*	**	*	*	*	7*	High
Margioula-Siarkou et al, 2013			*	*		*	*	*	5*	Moderate
Minsart et al, 2017			*	*	**	*	*	*	7*	High
Mozooni et al, 2020	*	*	*	*	**	*	*	*	9*	High

	SELECTION				COMPARABILITY	OUTCOME			Total scores	
**Author, Year**	Representativeness of the exposed cohort *	Selection of the non-exposed cohort *	Ascertainment of exposure *	No outcome of interest at the start of the study *	A: Study controls for age and/or BMI * B: Study controls for *other confounders	A: doctor's diagnosis OR objective measurements B: parent/self-reported doctor's diagnosis OR use of medication	Follow-up long enough for outcomes *	Adequacy of follow up of cohorts *		
Kragelund Nielsen et al, 2020	*	*	*	*	**	*	*	*	9*	High
Ozel et al, 2018				*	**	*	*	*	6*	Moderate
Paz-Zulueta et al, 2015	*	*	*	*	**	*	*	*	9*	High
Besharat Pour et al, 2014	*	*	*	*	**	*	*	*	9*	High
Racape et al, 2013	*	*	*	*	**	*	*	*	9*	High
Raimondi et al, 2013			*	*		*	*	*	5*	Moderate
Ray et al, 2014	*	*	*	*	**	*	*	*	9*	High
Reed et al, 2005	*	*	*	*		*	*	*	7*	High
Restrepo-Mesa et al, 2015	*	*	*	*	**	*	*	*	9*	High
Råssjö et al, 2013	*	*	*	*		*	*	*	7*	High
Scholaske et al, 2019	*	*	*	*	**	*	*	*	9*	High
Sdona et al, 2019			*	*	**	*	*	*	7*	High
Seghieri et al, 2020	*	*	*	*	**	*	*	*	9*	High
Shah et al, 2011			*	*	**	*	*	*	7*	High
Sole et al, 2018	*	*	*	*	**	*	*	*	9*	High
Sosta et al, 2008			*	*	**	*	*	*	7*	High
Turkay et al, 2020			*	*		*	*	5	5*	Moderate
Urquia et al, 2015	*	*	*	*	**	*	*	*	9*	High
Vahratian et la, 2004			*	*	**	*	*	*	7*	High
Walsh et al, 2011			*	*		*	*	*	5*	Moderate
Wanigaratne et al, 2018	*	*	*	*	**	*	*	*	9*	High
Xirasagar et al, 2011			*	*	**	*	*	*	7*	High
Yoong et al, 2004			*	*		*	*	*	5*	Moderate
Zanconato et al, 2011			*	*	*	*	*	*	6*	High
Zuppa et al, 2010			*	*		*	*	*	5*	Moderate

Supplementary table 4. Quality assessment of included studies using the Newcastle–Ottawa Quality Assessment Scale for cross-sectional studies.

	SELECTION				COMPARABILITY	OUTCOME		Total scores	Quality of study
**Author**	Representativeness of the samples	Sample size	Non-responders	Ascertainment of the exposure	A: study controls for age and/or BMI<br>B: control for any additional factor	Assessment of the outcome<br>a) Independent blind assessment.<br>b) Record linkage.<br>c) Self report.	Statistical test		
Abdulrahim et al. 2018	*	*	*	*	**	**	*	9*	High
Agbemenu et al, 2019	*	*	*	*		**	*	7*	High
Akselsson et al, 2020	*	*	*	*	**	**	*	9*	High
Almeida et al, 2014		*	*	*		**	*	6*	Moderate
Bakken et al, 2015	*	*	*	*	**	**	*	9*	High
Bakken et al, 2017		*		*	**	**	*	7*	High
Bastola et al, 2020	*	*	*	*	**	**	*	9*	High
Bastola et al, 2019	*	*	*	*	**	**	*	9*	High
Bastola et al, 2020	*	*	*	*	**	**	*	9*	High
Belihu et al, 2016	*	*	*	*	**	**	*	9*	High
Belihu et al, 2017	*	*	*	*	**	**	*	9*	High
Belihu et al, 2017	*	*	*	*	**	**	*	9*	High
Bernis et al, 2013		*	*	*	**	**	*	8*	High
Bozorgmehr et al, 2018		*	*	*	**	**	*	8*	High
Breckenkamp et al, 2019		*	*	*	**	**	*	8*	High
Calderon-Margalit et al, 2015		*	*	*	**	**	*	7*	High
Castello et al, 2012	*	*	*	*	**	**	*	9*	High
Dahlen et al, 2013	*	*	*	*	**	**	*	9*	High
David et al, 2017		*	*	*	**	**	*	8*	High
Erenel et al, 2017				*	**	**	*	6*	Moderate
Eskild et al, 2020	*	*	*	*	**	**	*	9*	High
Fu et al, 2010	*	*	*	*	**	**	*	9*	High
Gagnon et al, 2013	*	*	*	*		**	*	7*	High
Garcia-Subirat et al, 2011	*	*	*	*		**	*	7*	High
Gillet et al, 2014	*	*	*	*	**	**	*	9*	High
Gould et al, 2003	*	*	*	*		**	*	7*	High
Hamilcikan and Can, 2017		*	*	*		**	*	6*	Moderate
Harding et al, 2006	*	*	*	*	**	**	*	9*	High
Hessol and Fuentes-Afflick, 2014	*	*	*	*	**	**	*	9*	High
Hsieh et al, 2011	*	*	*	*	**	**	*	9*	High
Juárez et al, 2019	*	*	*	*	**	**	*	9*	High
Juárez and Revuelta-Eugercios, 2014	*	*	*	*	**	**	*	9*	High
Juarez et al, 2017	*	*	*	*	**	**	*	9*	High
Juárez et al, 2018	*	*	*	*	**	**	*	9*	High
Kana et al, 2019	*	*	*	*	**	**	*	9*	High
Kane et al, 2018	*	*	*	*	**	**	*	9*	High
Khanolkar et al, 2015	*	*	*	*	**	**	*	9*	High
Krishnakumar et al, 2011	*	*	*	*	**	**	*	9*	High
Liu et al, 2008	*	*	*	*	**	**	*	9*	High
Ma and Bauman, 1996	*	*	*	*		**	*	7*	High
Madan et al, 2006	*	*	*	*		**	*	7*	High
Malamitsi-Puchner et al, 1994			*	*		**	*	5*	Moderate
Malin and Gissler, 2009	*	*	*	*	**	**	*	9*	High
Moore et al, 2009	*	*	*	*	**	**	*	9*	High
Naimy et al, 2013	*	*	*	*	**	**	*	9*	High
Naimy et al, 2015	*	*	*	*	**	**	*	9*	High
Nilsen et al, 2018	*	*	*	*	**	**	*	9*	High
Opondo et al, 2020	*	*	*	*	**	**	*	9*	High
Ortiz et al, 2019			*	*		**	*	5*	Moderate
Park et al, 2015	*	*	*	*	**	**	*	9*	High
Pedersen et al, 2012	*	*	*	*	**	**	*	9*	High
Racape et al, 2016	*	*	*	*	**	**	*	9*	High
Reeske et al, 2012	*	*	*	*	**	**	*	9*	High
Reiss et al, 2015		*	*	*	**	**	*	8*	High
Sanchalika and Teresa, 2015	*	*	*	*	**	**	*	9*	High

Song et al, 2017	*	*	*	*	**	**	*	9*	High
Sørbye et al, 2015	*	*	*	*	**	**	*	9*	High
Sørbye et al, 2014	*	*	*	*	**	**	*	9*	High
Sow et al, 2018	*	*	*	*	**	**	*	9*	High
Sow et al, 2019	*	*	*	*	**	**	*	9*	High
Sunil et al, 2012	*	*	*	*	**	**	*	9*	High
Teixeir et al, 2013		*	*	*	**	**	*	8*	High
Tsimbos and Verropoulou, 2011	*	*	*	*		**	*	7*	High
Urquia et al, 2010	*	*	*	*	**	**	*	9*	High
Urquia, 2015, Canada data	*	*	*	*	**	**	*	9*	High
Vangen et al, 2002	*	*	*	*	**	**	*	9*	High
Vangen et al, 2000	*	*	*	*	**	**	*	9*	High
Verschuuren et al, 2020			*	*		**	*	5*	Moderate
Vetter et al, 2013	*	*	*	*	**	**	*	9*	High
Vik et al, 2019	*	*	*	*	**	**	*	9*	High
Vik et al, 2020	*	*	*	*	**	**	*	9*	High
Villadsen et al, 2010	*	*	*	*	*	**	*	8*	High
Wanigaratne et al, 2016	*	*	*	*	**	**	*	9*	High
Zeitlin et al, 2011	*	*	*	*	**	**	*	9*	High

Supplementary table 5: The results of meta-regression assessing the association between maternal age and the risk of adverse pregnancy outcomes in immigrant women compared to those with the native origin background.

Outcome	P-value obtained from Meta-regression
Macrosomia	0.088
SGA	0.440
LGA	**0.001**
Birth trauma	0.790
Apgar <7	0.670
Labor induction	0.872
Instrumental delivery	0.617
Cesarean section	0.675
Emergency caesarean section	0.906
Preeclampsia	0.561
PIH	0.838
Preterm Birth	0.520
Oligohydramnios	0.753
GDM	**0.015**

GDM: gestational diabetes mellitus; PIH: pregnancy-induced hypertension; LGA: large for gestational age; SGA: small for gestational age.

Supplementary figure 1. Forest plot of the pooled prevalence of macrosomia (more than 4000 gr) in the immigrant and native origin population.

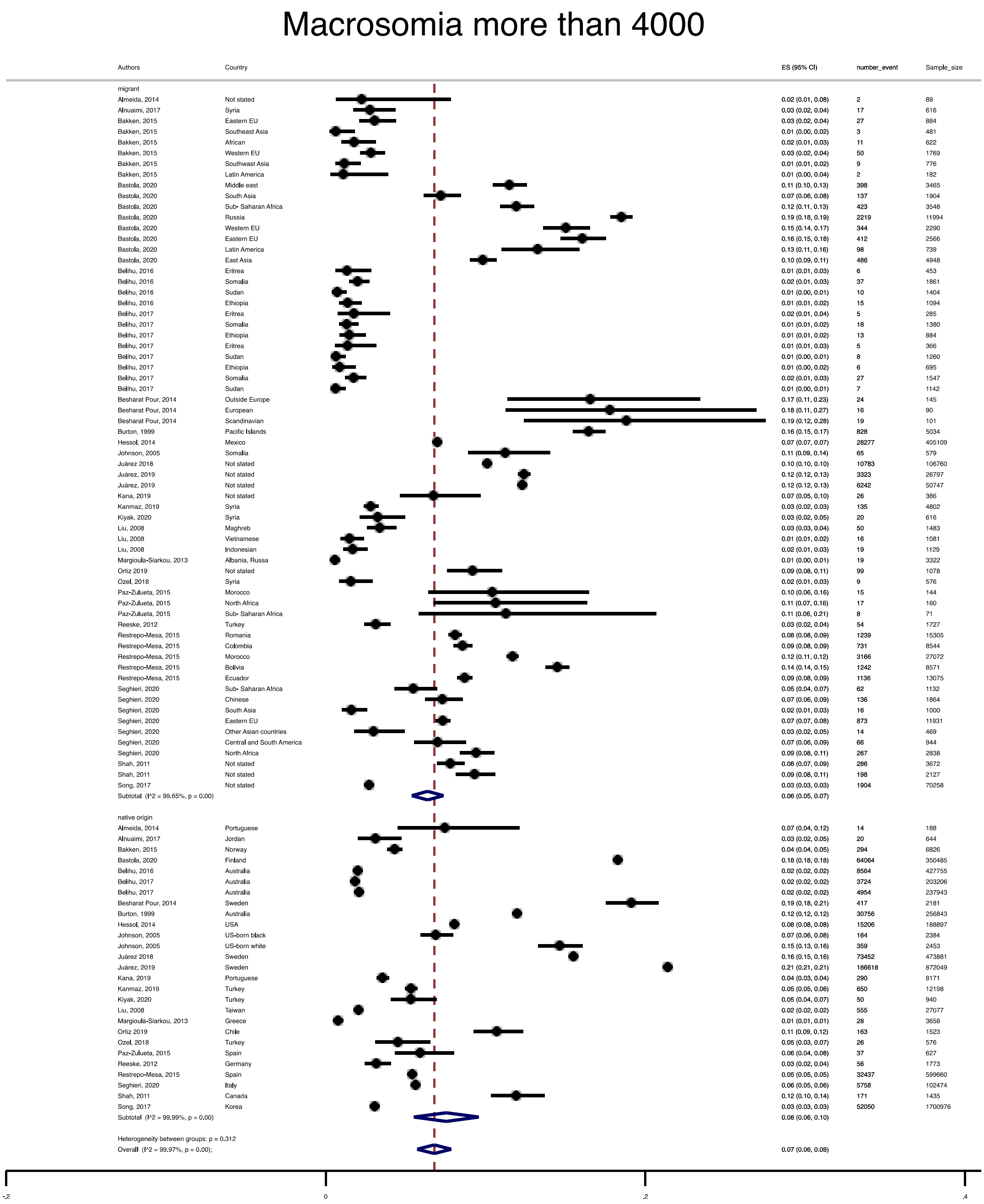

Supplementary figure 2. Forest plot of the pooled prevalence of small for gestational age (SGA) in the immigrant and native origin population.

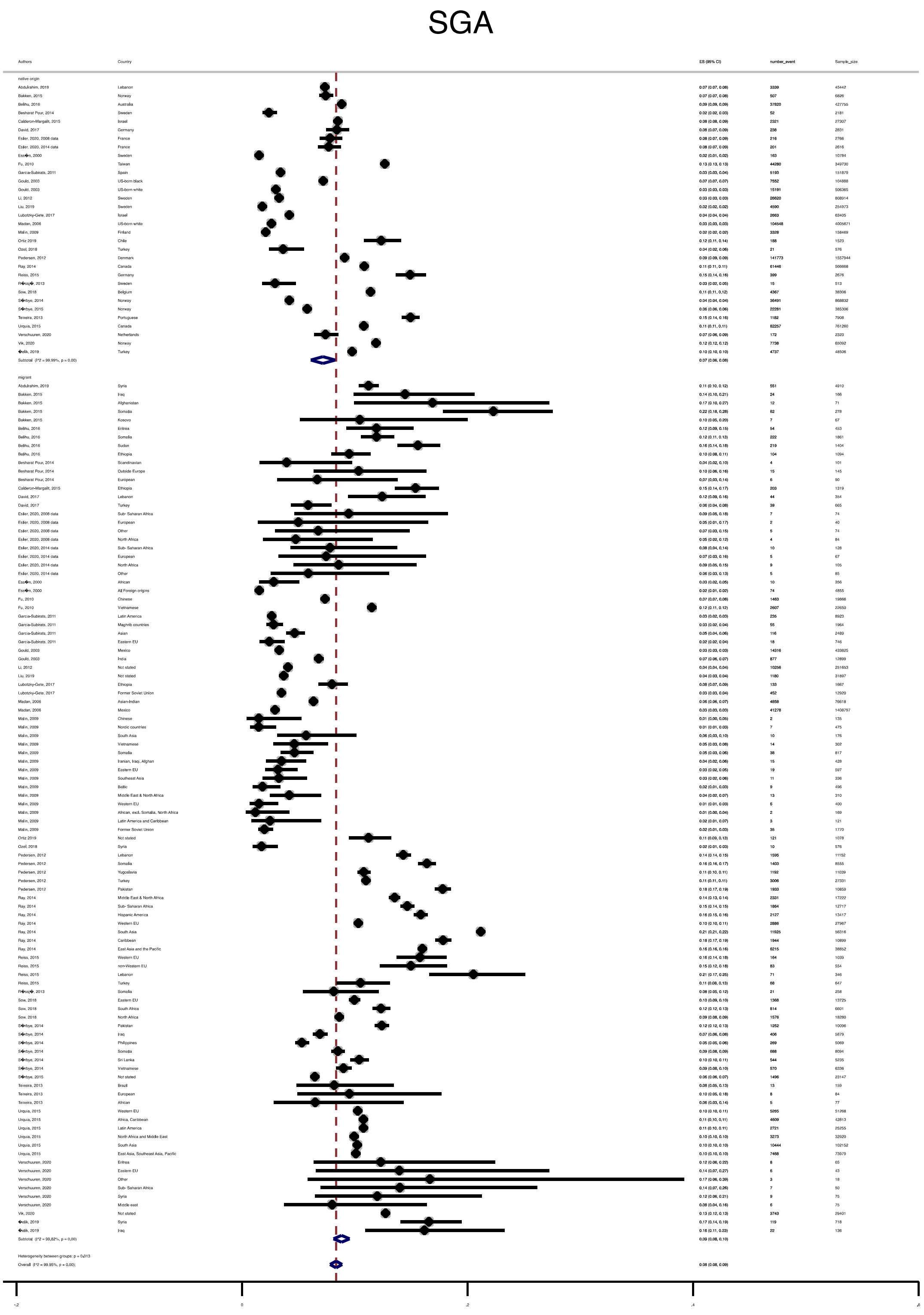

Supplementary figure 3. Forest plot of the pooled prevalence of large for gestational age (LGA) in the immigrant and native origin population.

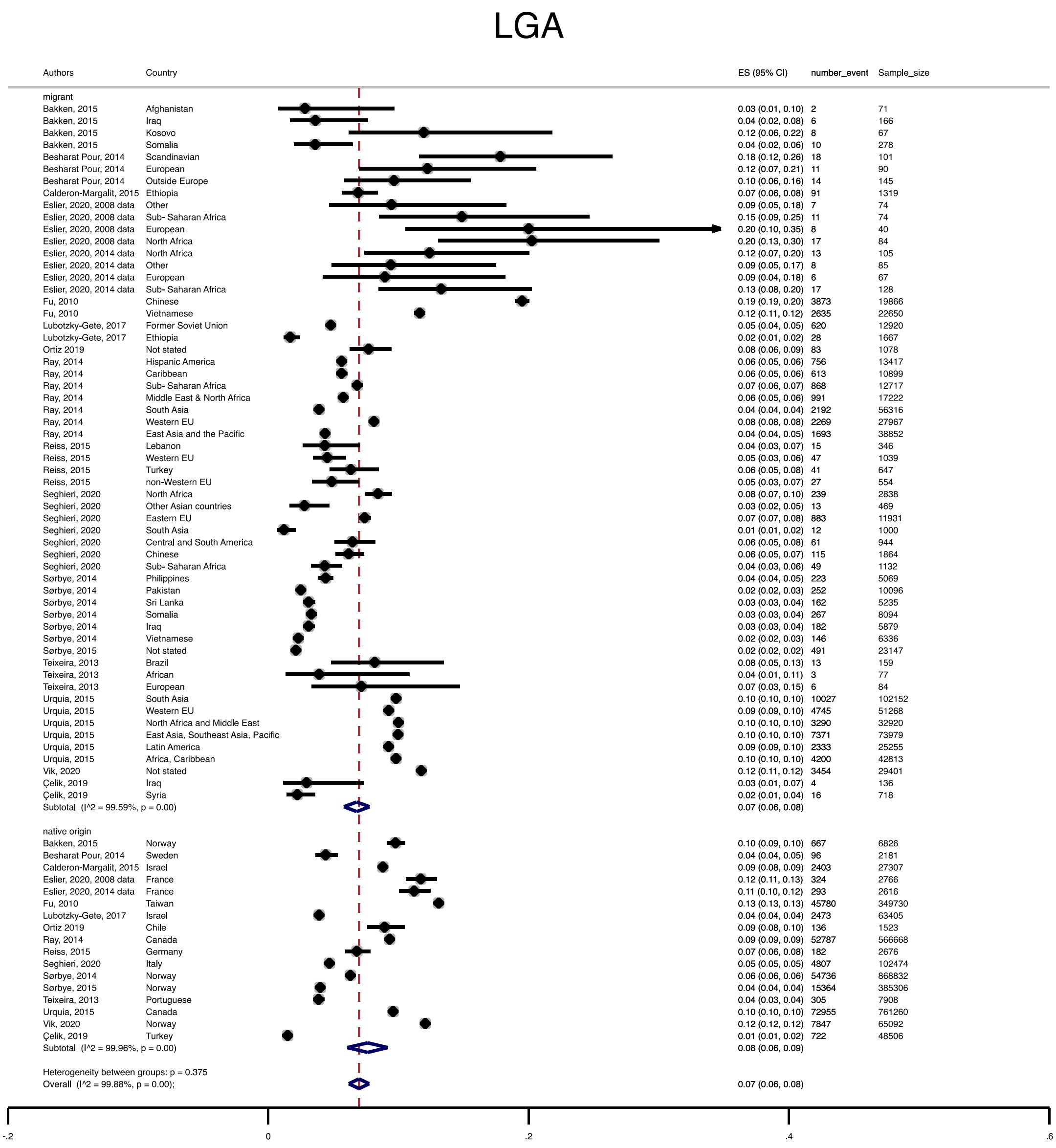

Supplementary figure 4. Forest plot of the pooled prevalence of birth trauma in the immigrant and native origin population.

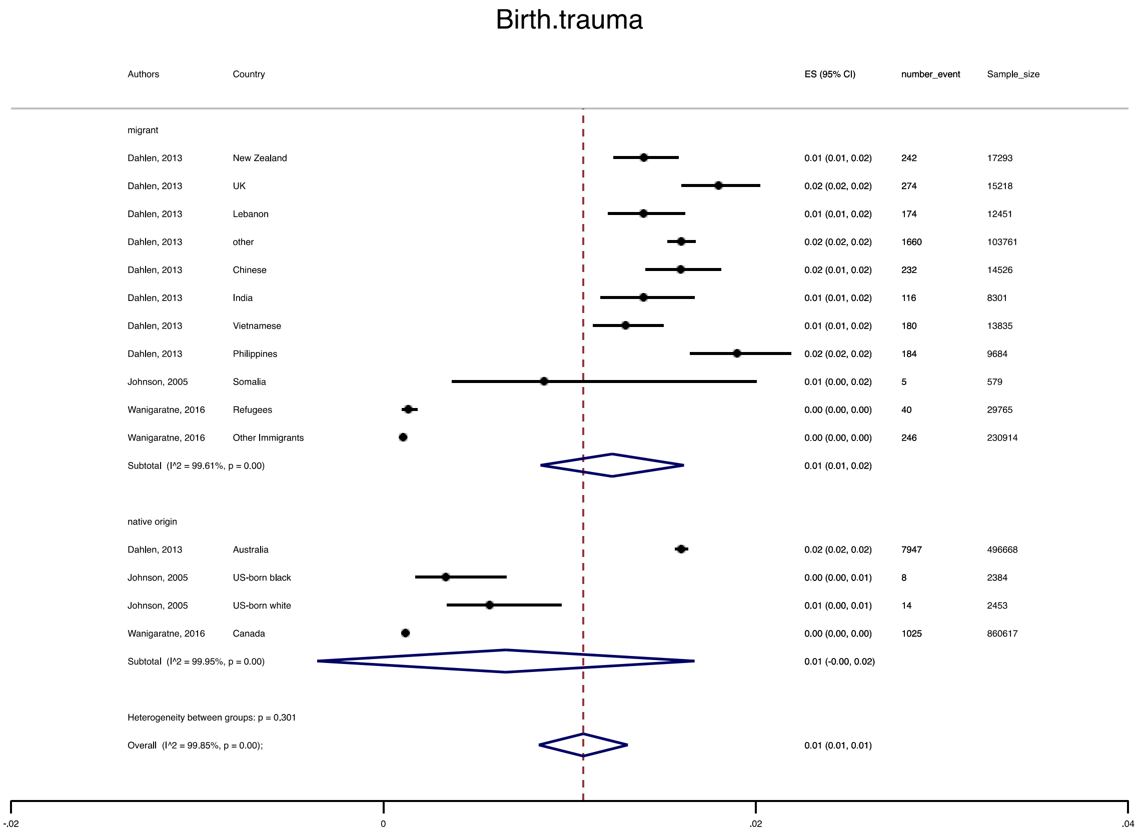

Supplementary figure 5. Forest plot of the pooled prevalence of admission to the neonatal intensive care unit (NICU) in the immigrant and native origin population.

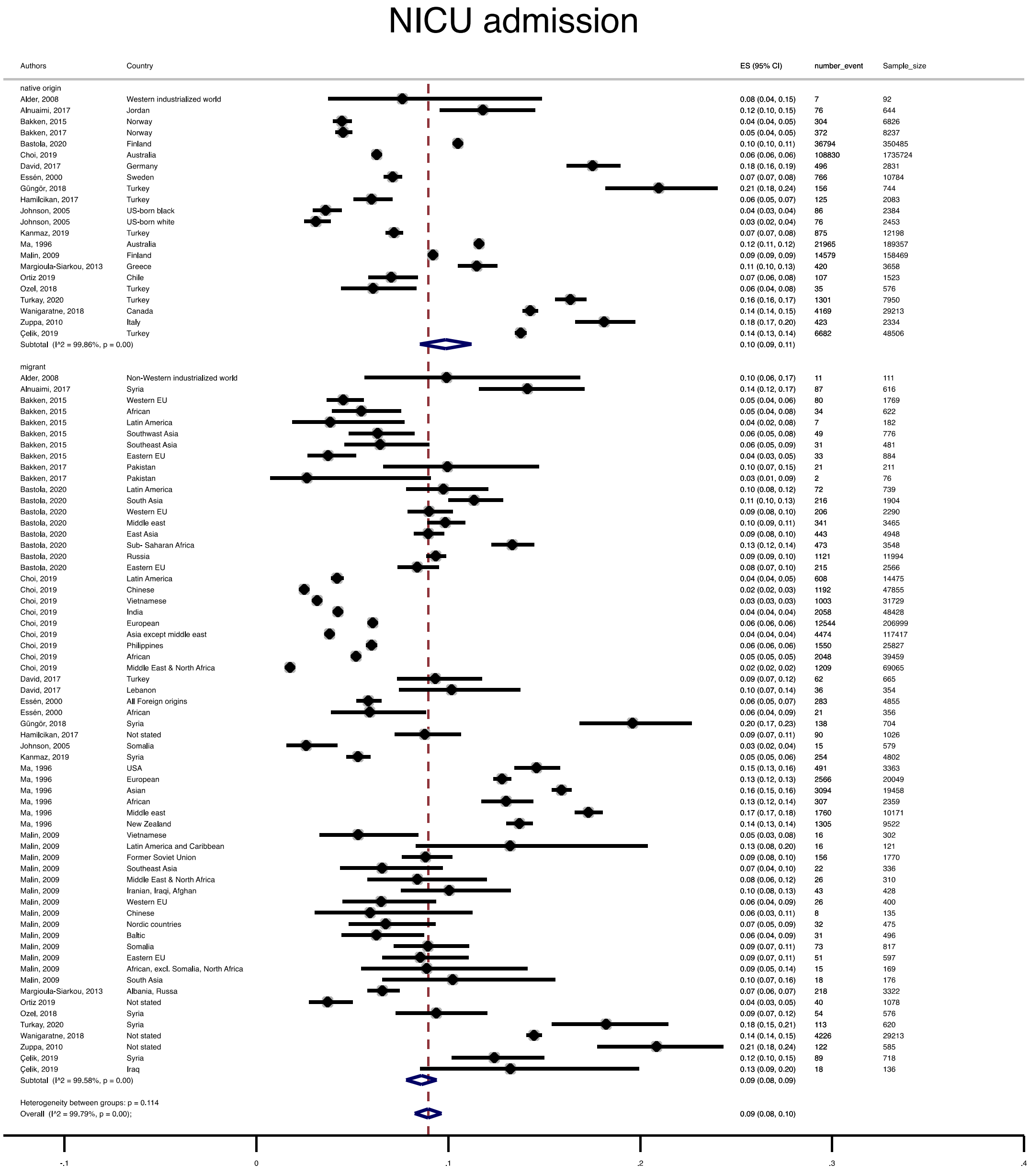

Supplementary figure 6. Forest plot of the pooled prevalence of respiratory distress syndrome (RDS) in the immigrant and native origin population.

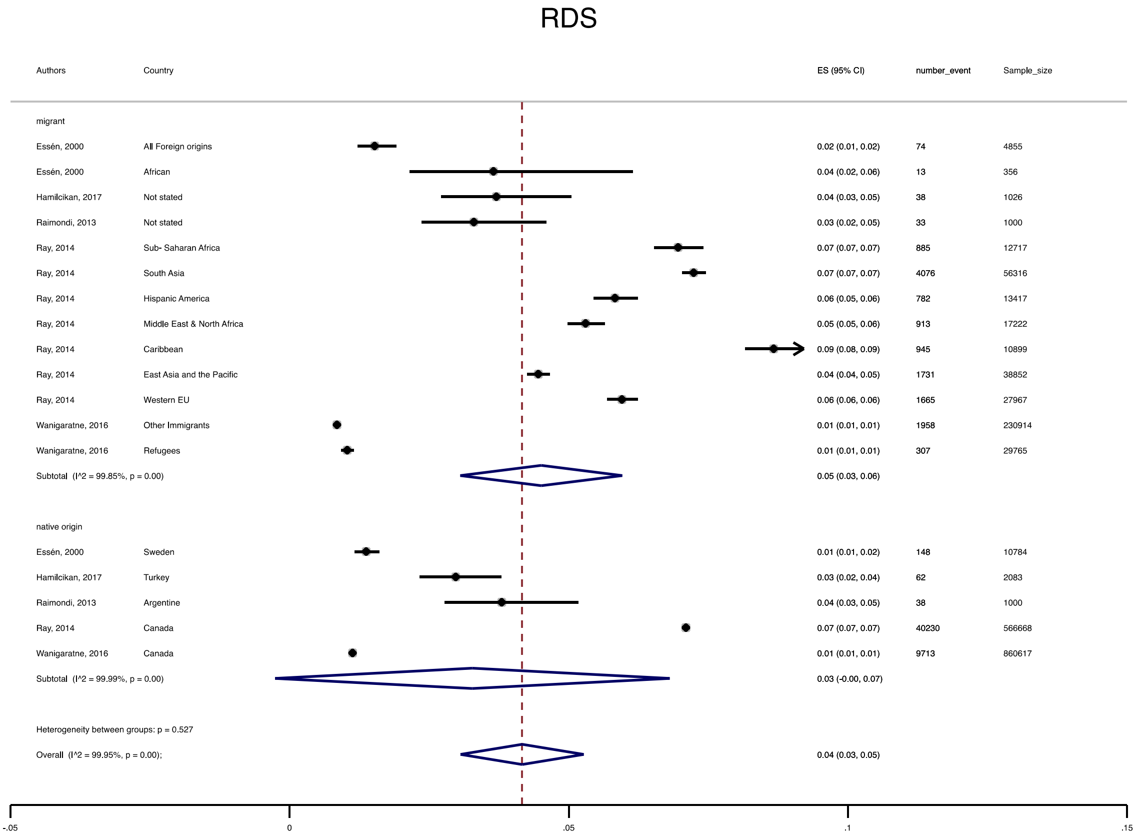

Supplementary figure 7. Forest plot of the pooled prevalence of Apgar score less than 7 in the immigrant and native origin population.

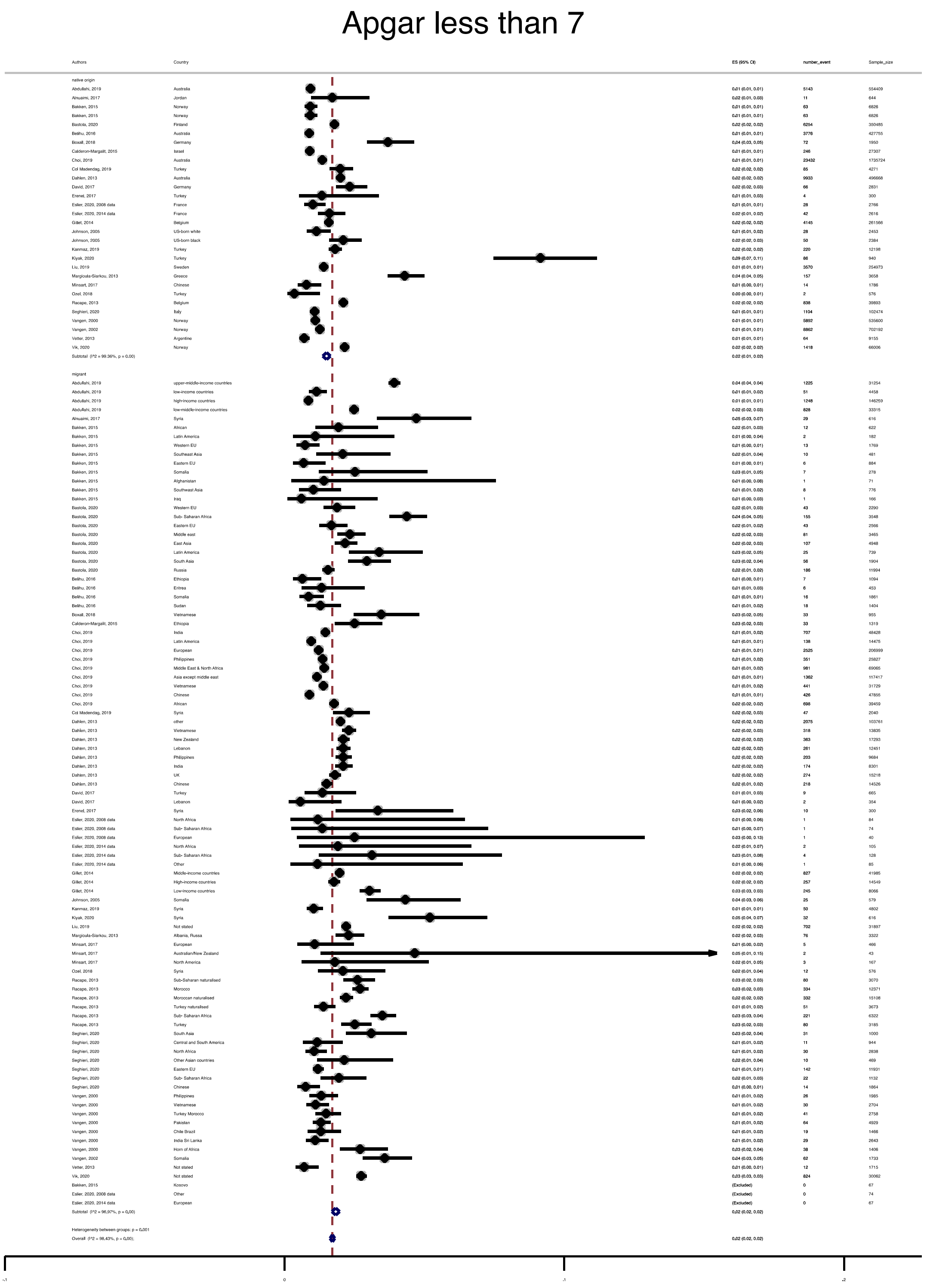

Supplementary figure 8. Forest plot of the pooled prevalence of labor induction in the immigrant and native origin population.

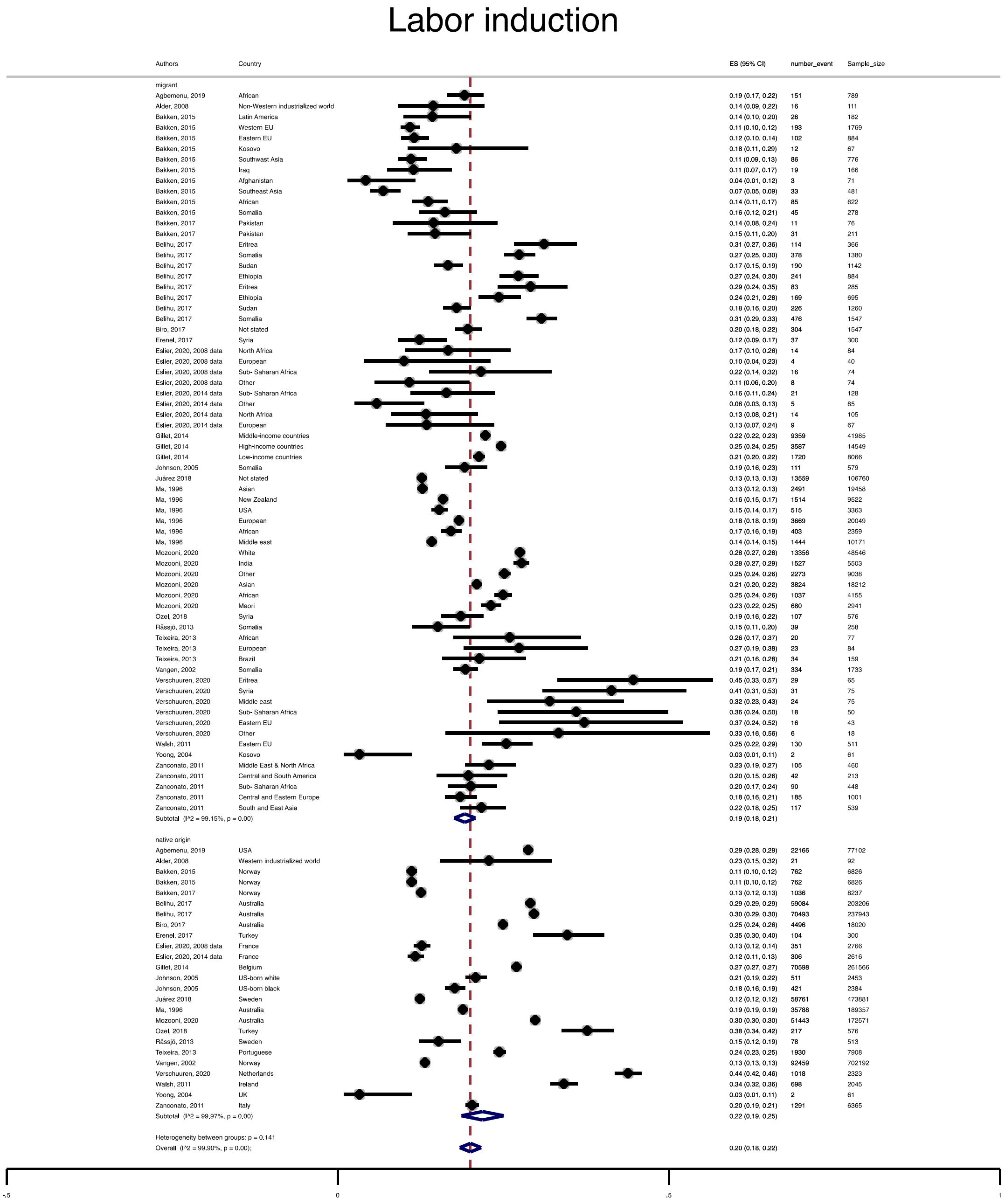

Supplementary figure 9. Forest plot of the pooled prevalence of instrumental delivery in the immigrant and native origin population.

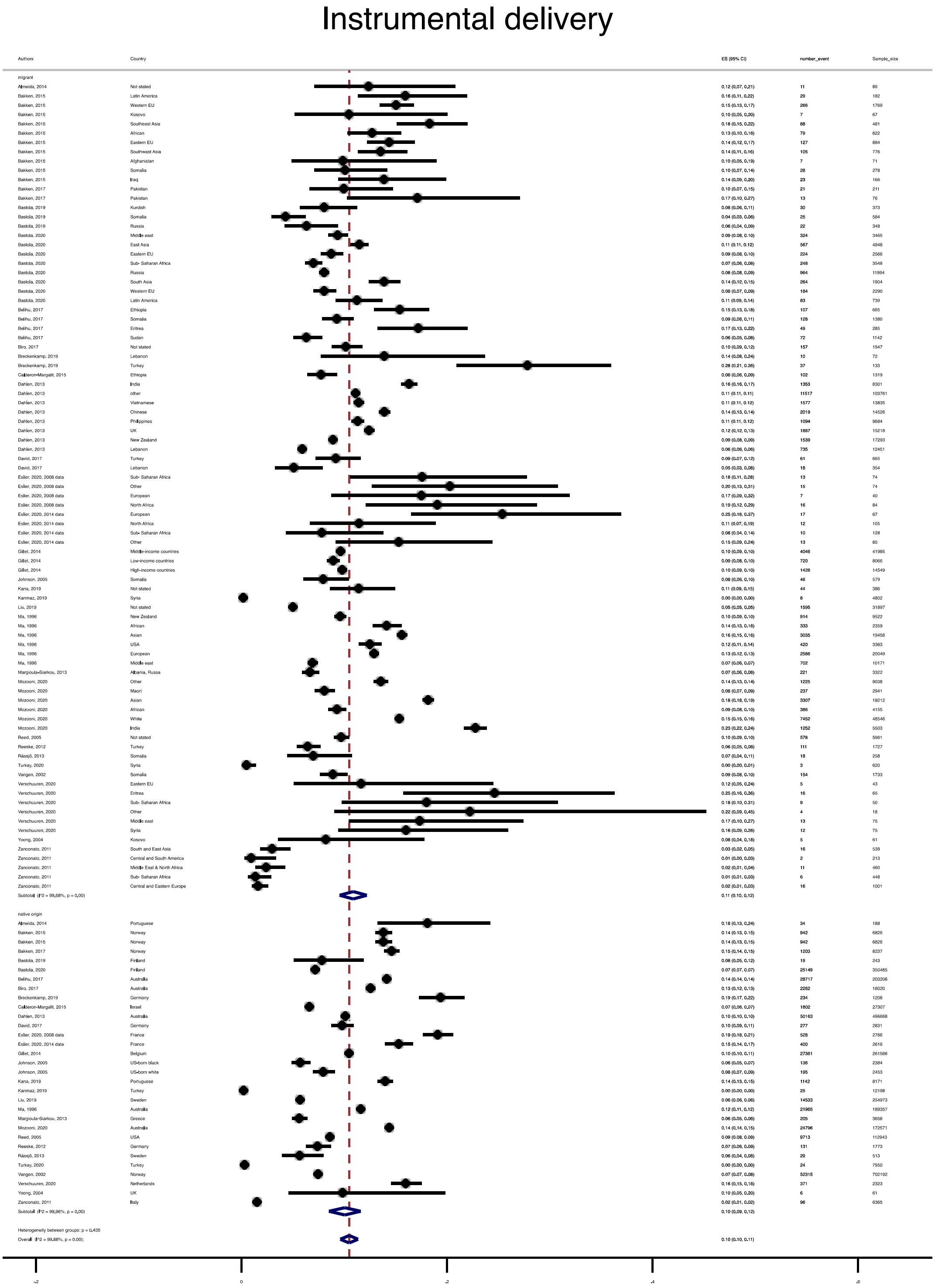

Supplementary figure 10. Forest plot of the pooled prevalence of cesarean section in the immigrant and native origin population.

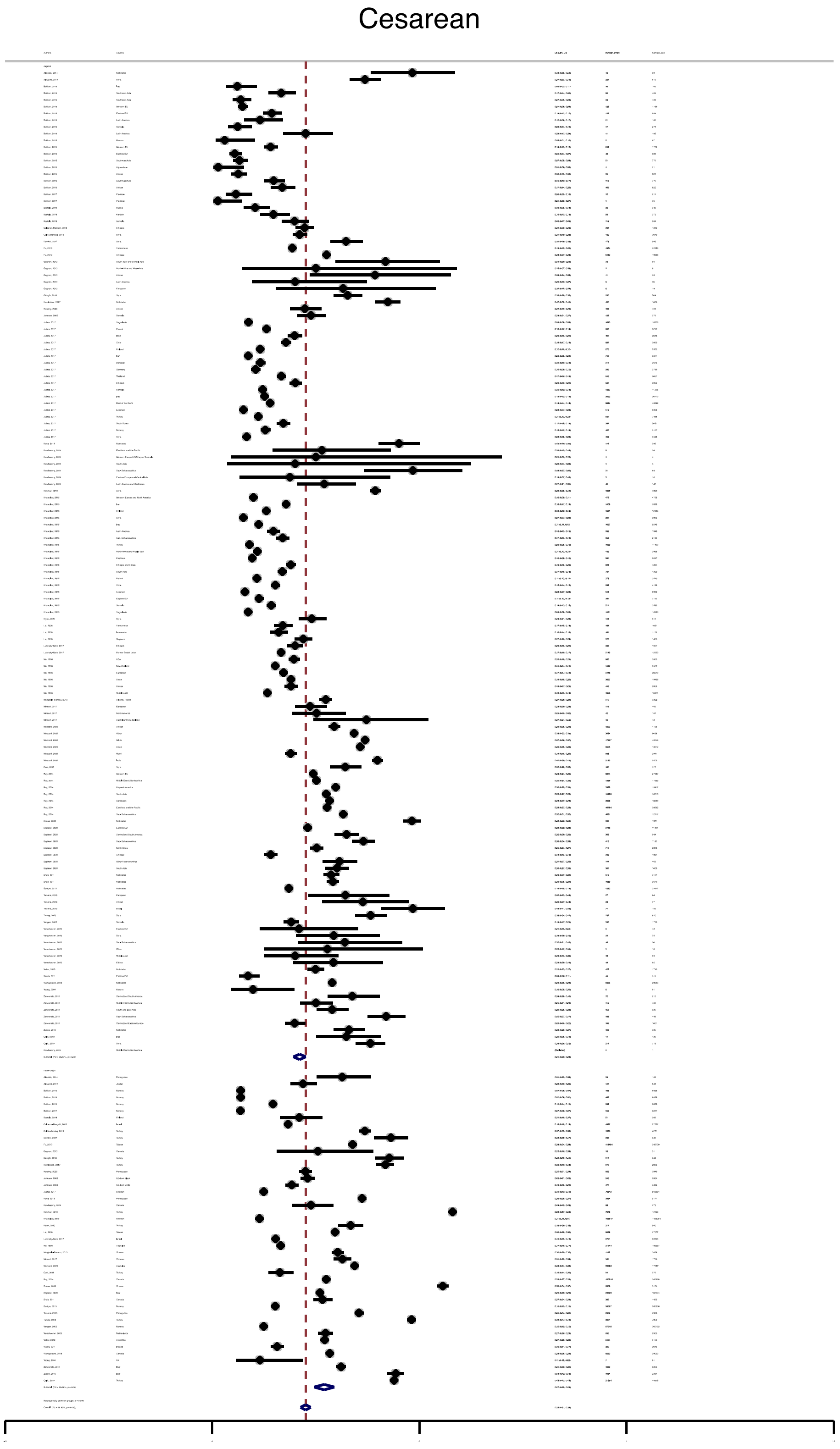

Supplementary figure 11. Forest plot of the pooled prevalence of emergency cesarean section in the immigrant and native origin population.

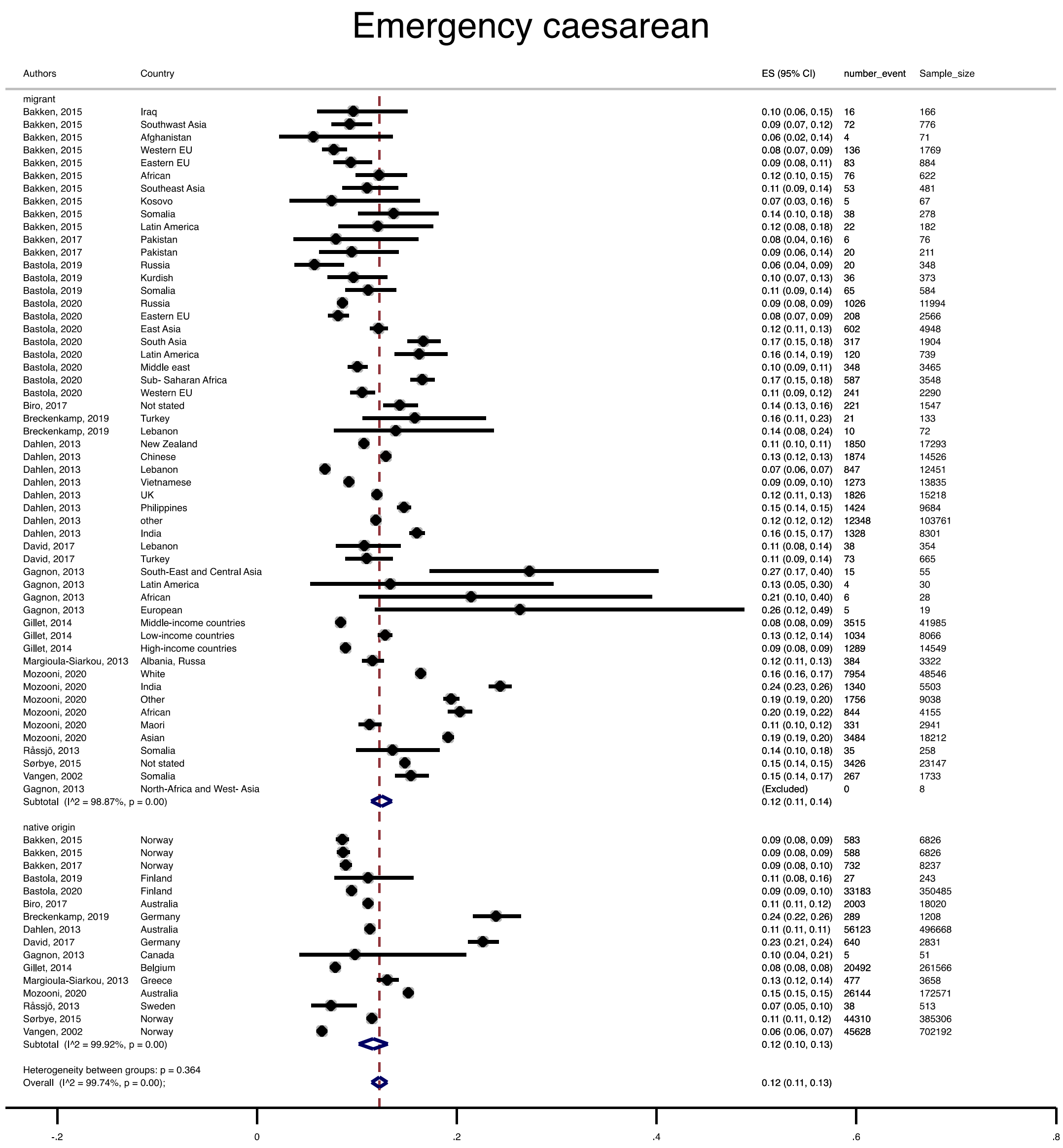

Supplementary figure 12. Forest plot of the pooled prevalence of shoulder dystocia in the immigrant and native origin population.

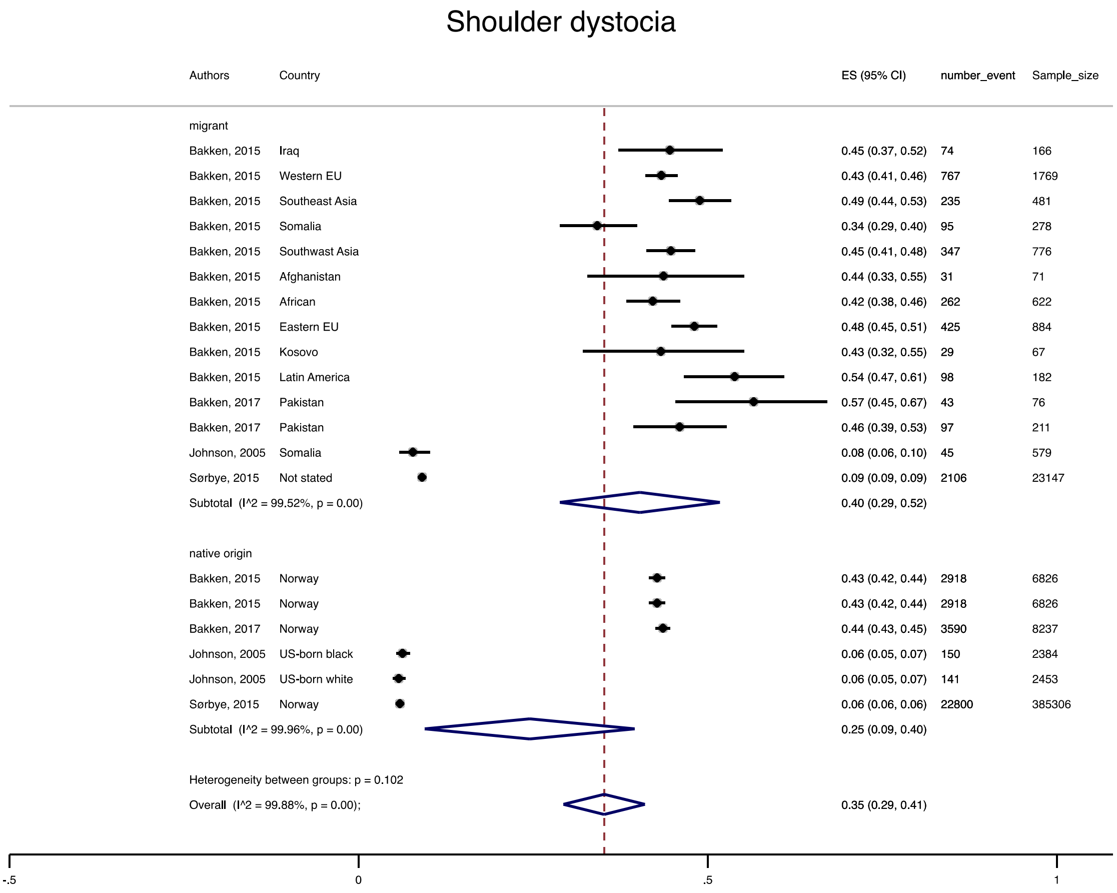

Supplementary figure 13. Forest plot of the pooled prevalence of preeclampsia in the immigrant and native origin population.

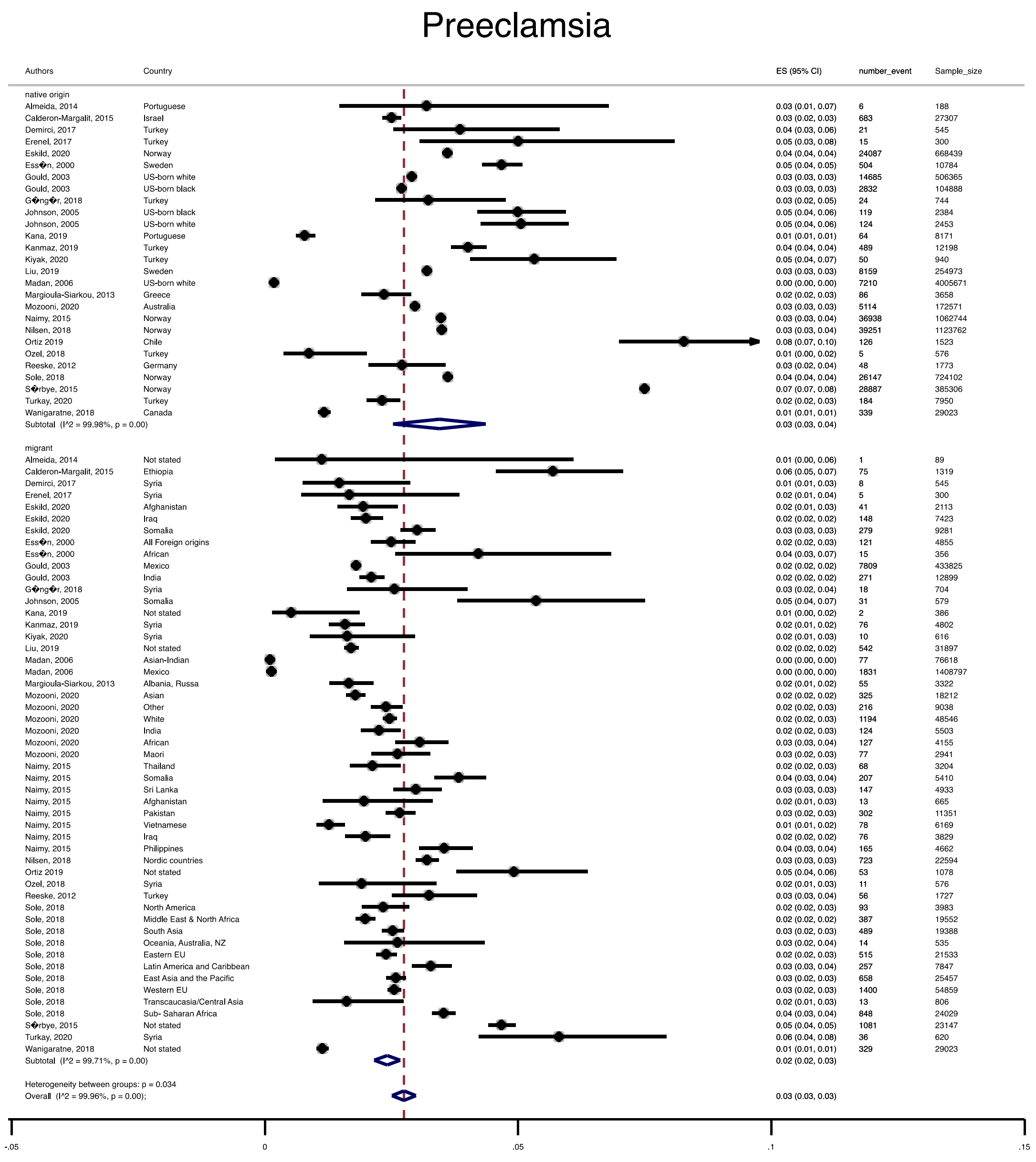

Supplementary figure 14. Forest plot of the pooled prevalence of pregnancy induced hypertension (PIH) in the immigrant and native origin population.

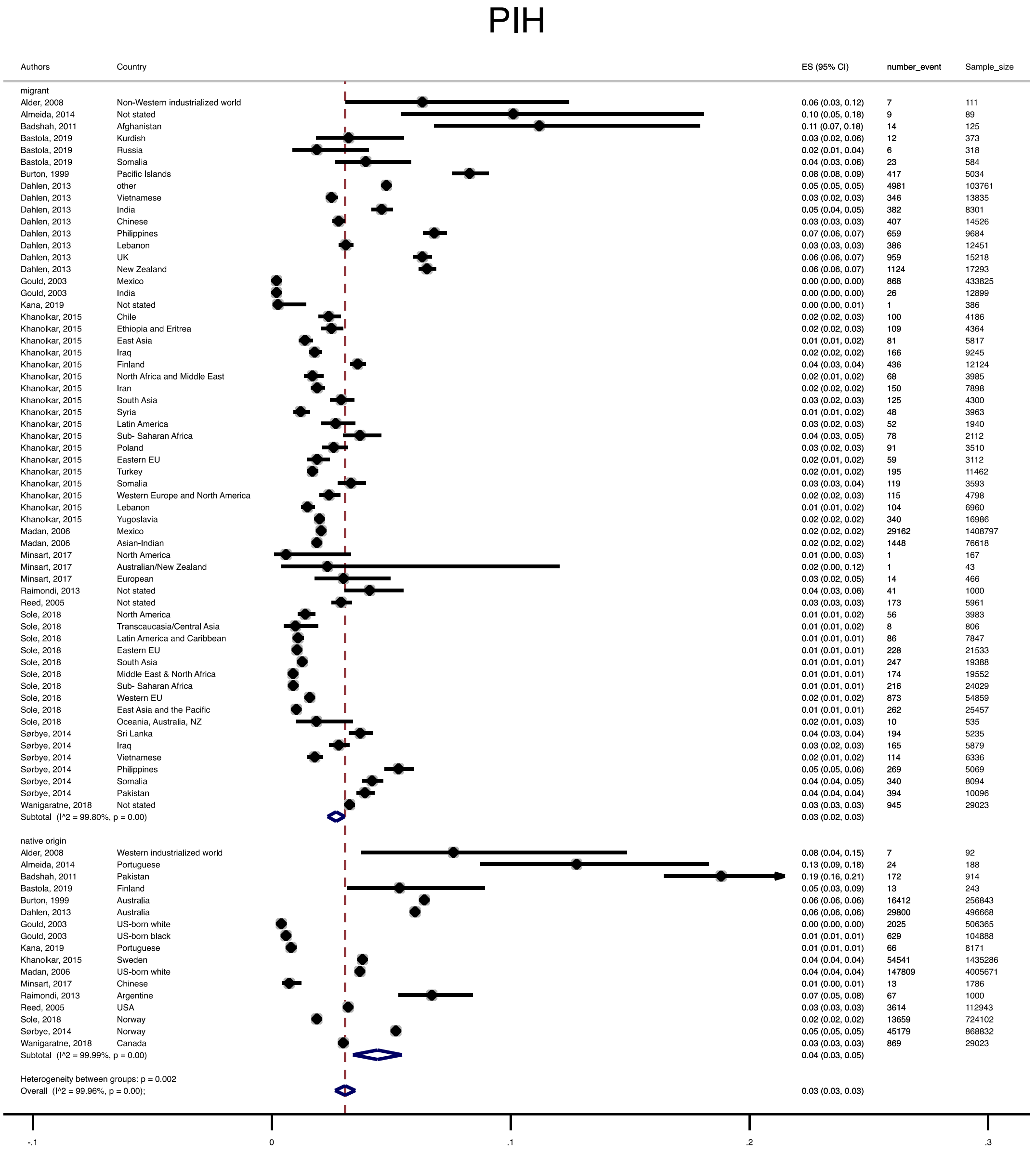

Supplementary figure 15. Forest plot of the pooled prevalence of preterm birth in the immigrant and native origin population.

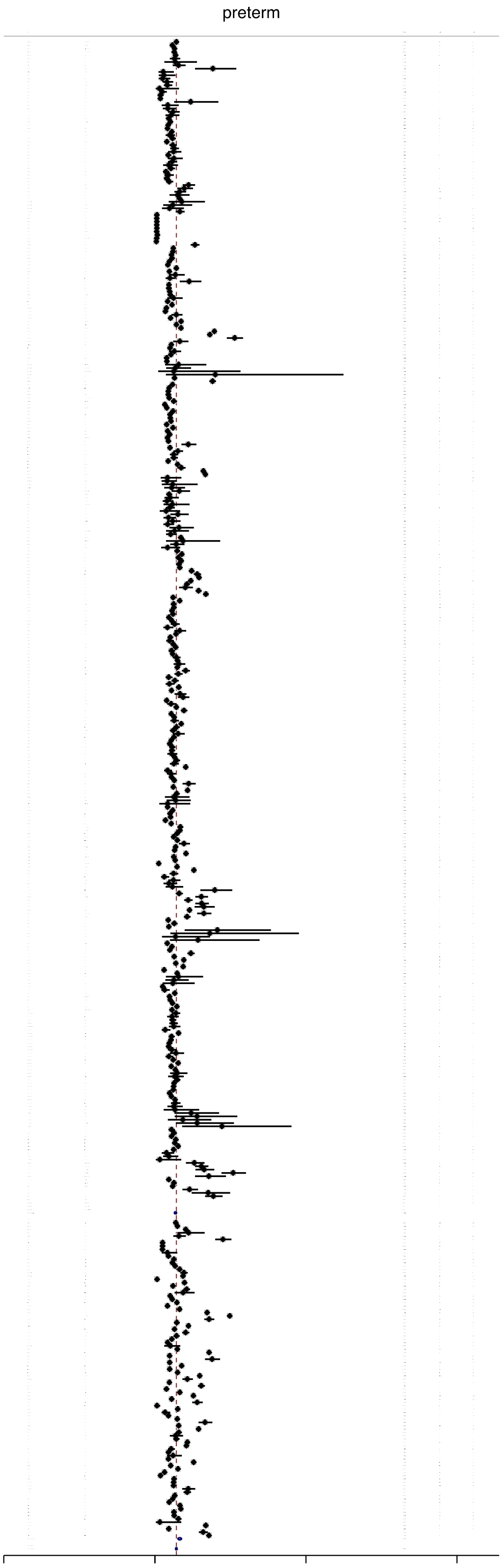

Supplementary figure 16. Forest plot of the pooled prevalence of primary cesarean section in the immigrant and native origin population.

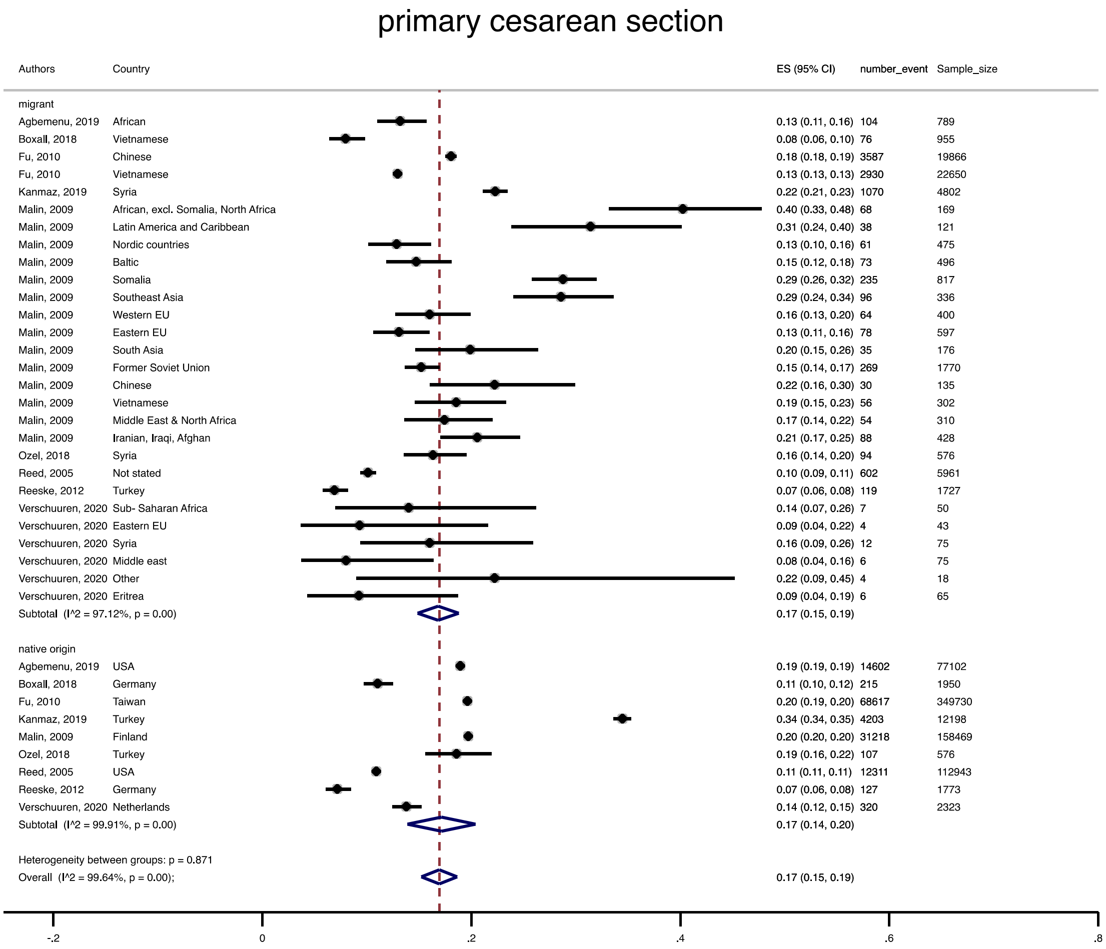

Supplementary figure 17. Forest plot of the pooled prevalence of gestational diabetes mellites (GDM) in the immigrant and native origin population.

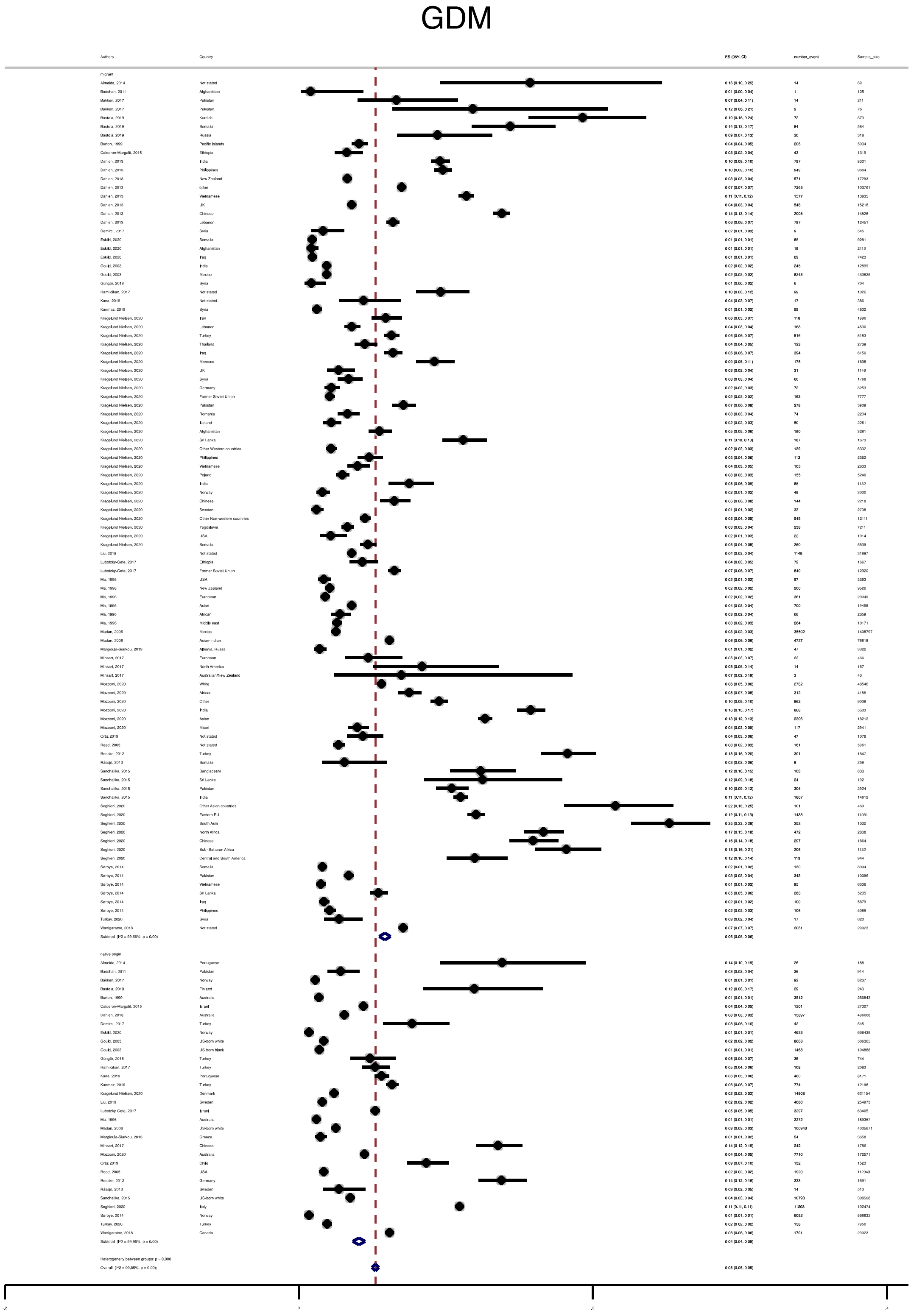

Supplementary figure 18. Forest plot of the pooled odds ratio of cesarean section in the immigrant and native origin population.

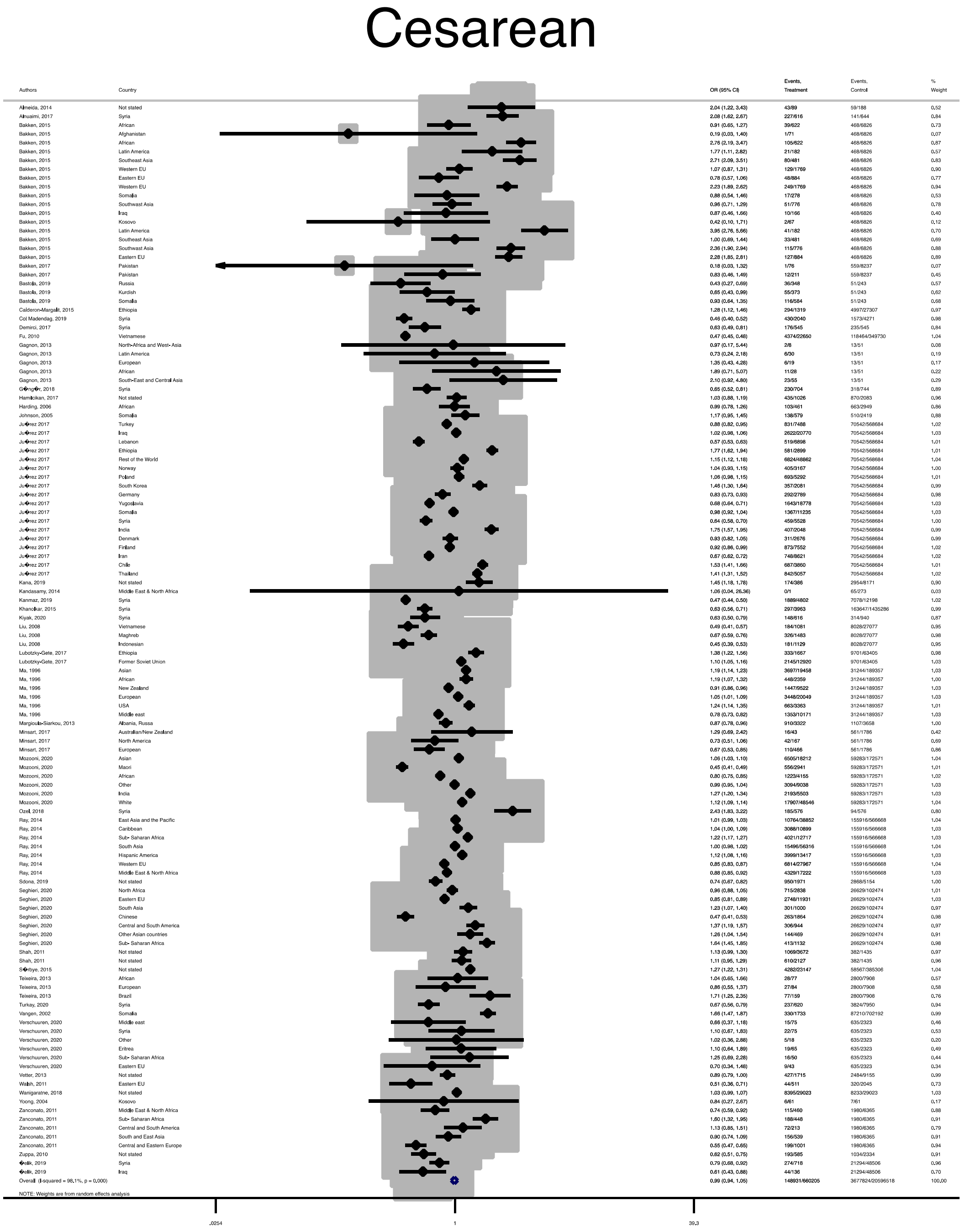

Supplementary figure 19. Forest plot of the pooled odds ratio of primary cesarean section (CS) in the immigrant and native origin population.

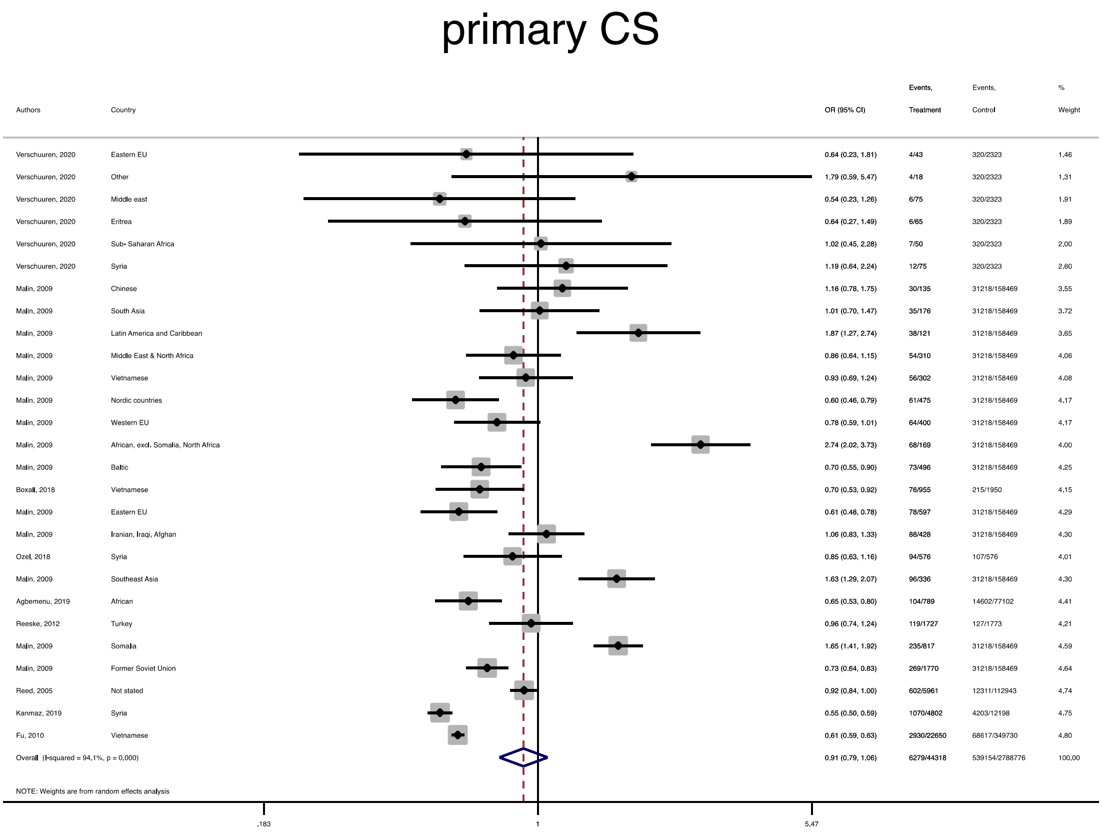

Supplementary figure 20. Forest plot of the pooled odds ratio of instrumental delivery in the immigrant and native origin population.

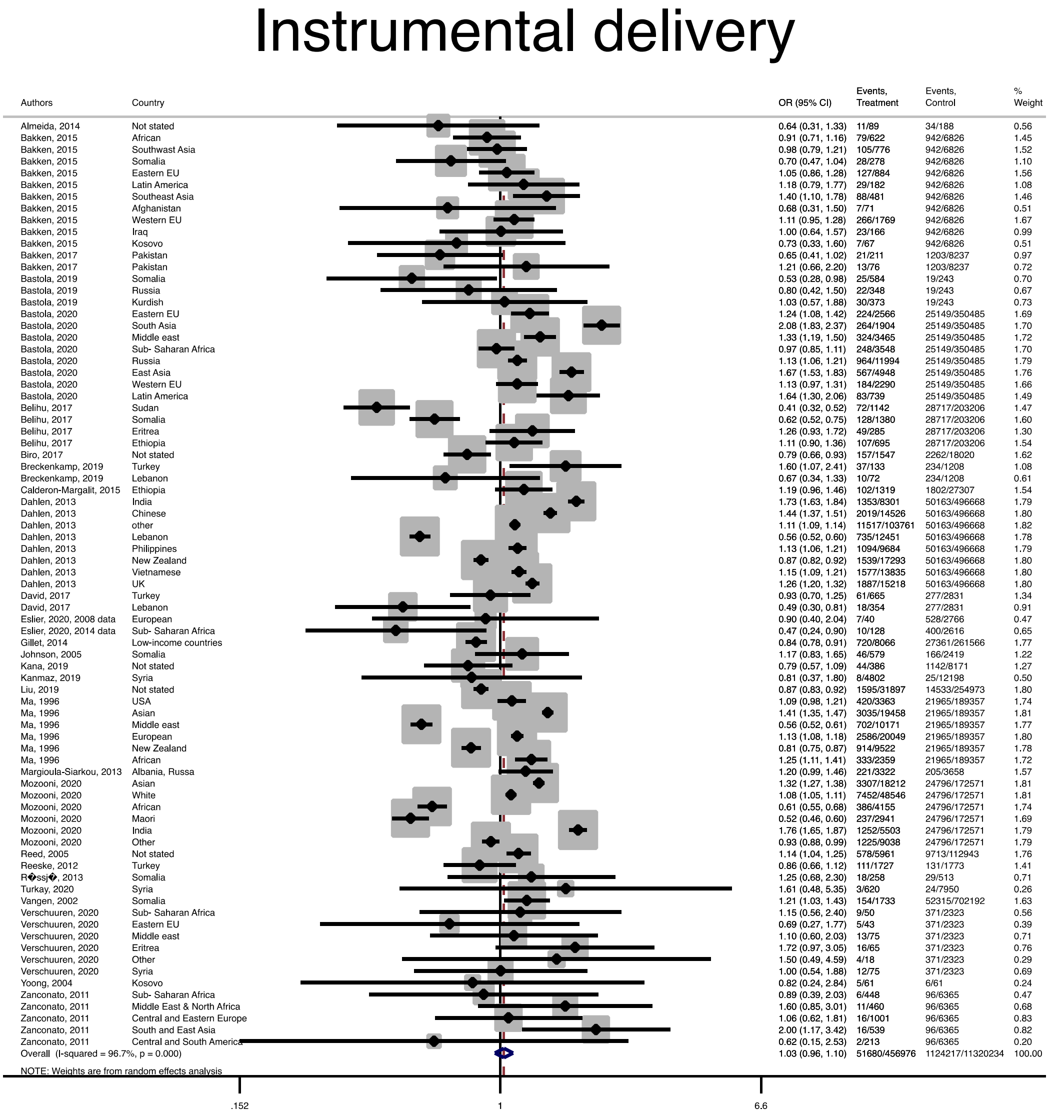

Supplementary figure 21. Forest plot of the pooled odds ratio of preterm birth in the immigrant and native origin population.

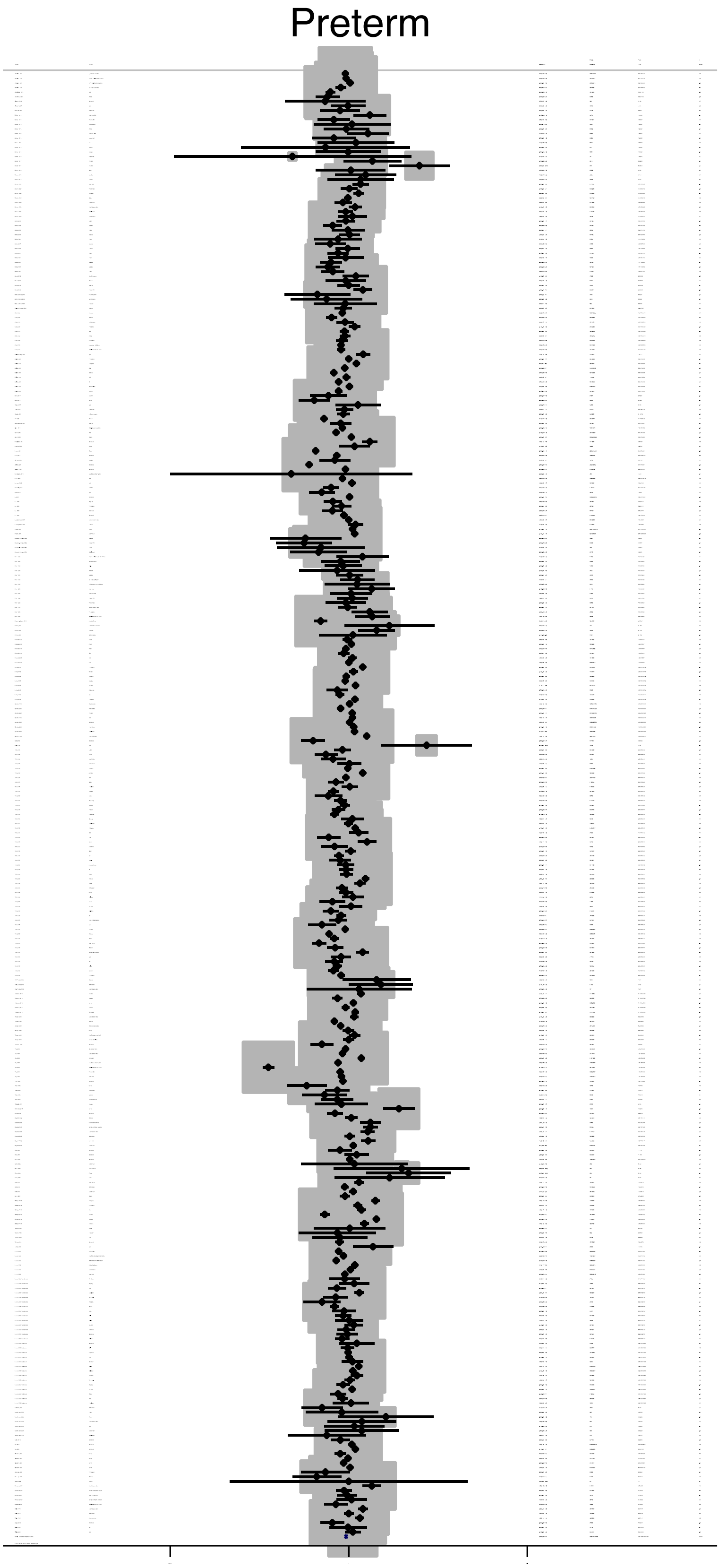

Supplementary figure 22. Forest plot of the pooled odds ratio of birth trauma in the immigrant and native origin population.

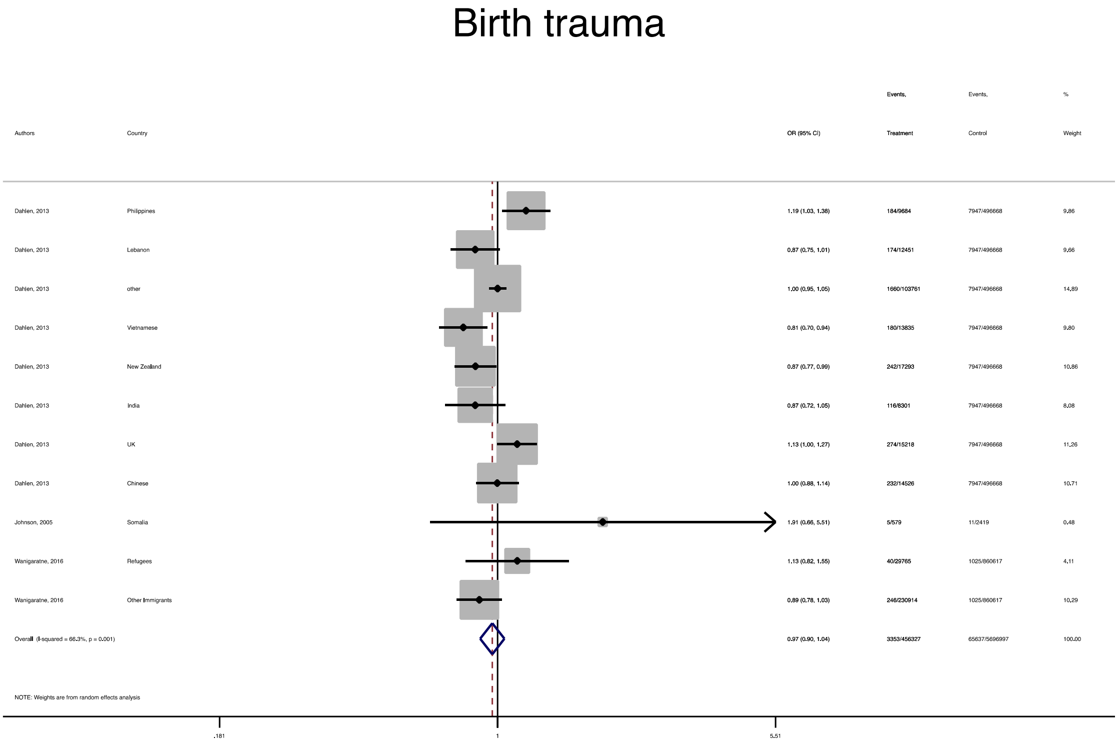

Supplementary figure 25. Bubble plot of the meta-regression relationships between the adverse pregnancy outcomes and geographical region of origin.

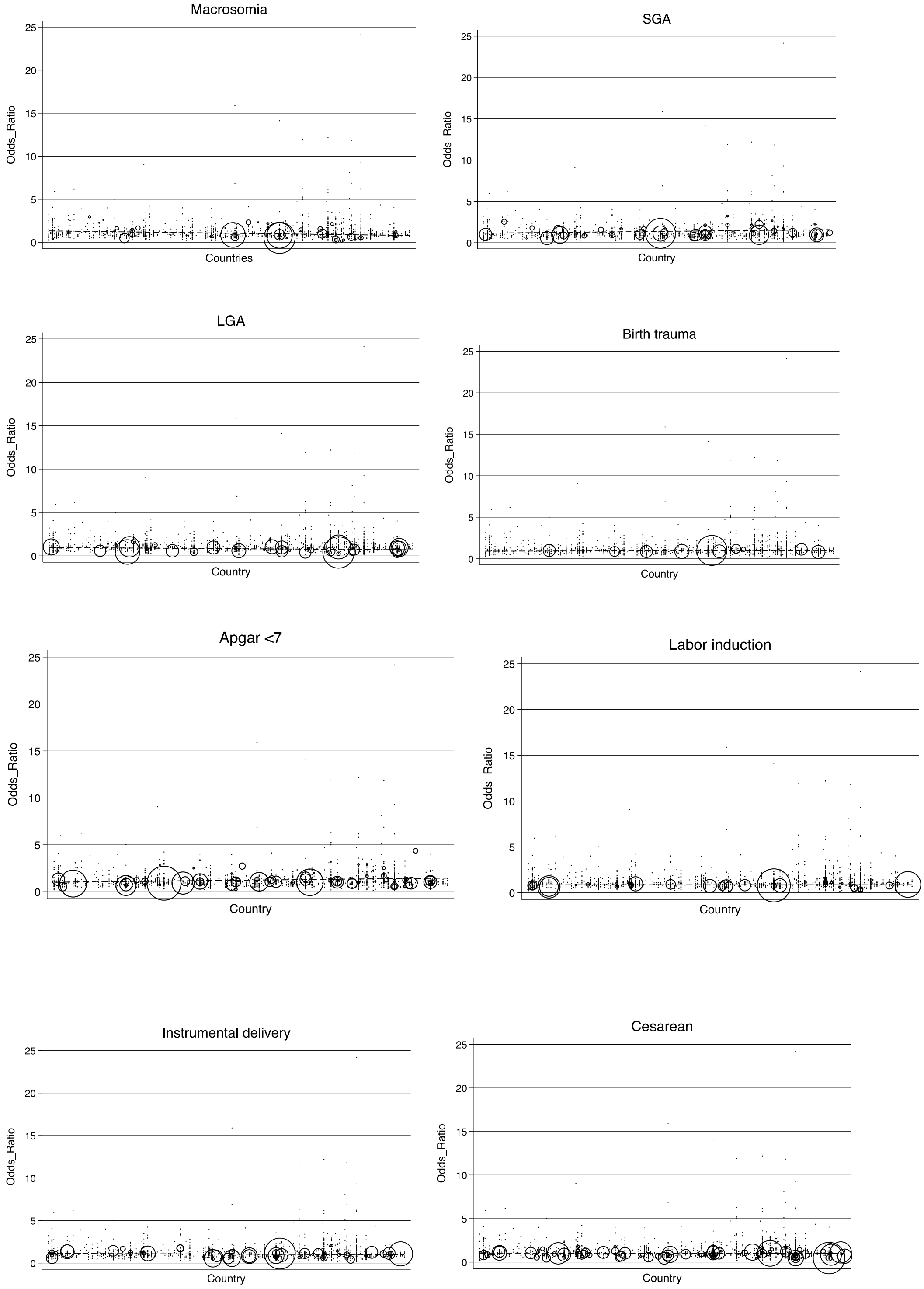

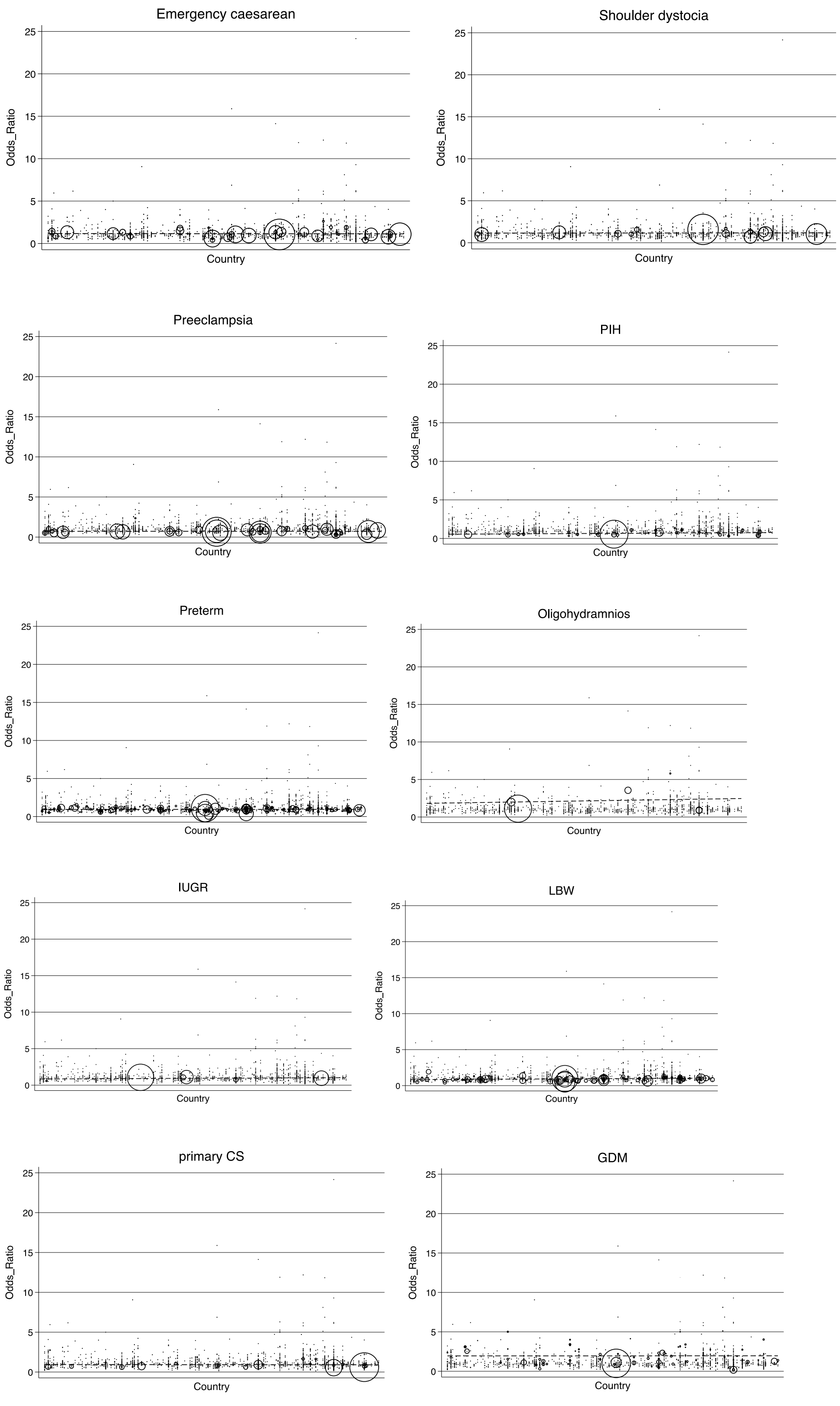
Emergency caesarean
Shoulder dystocia
Preeclampsia
PIH
Preterm
Oligohydramnios
IUGR
LBW
primary CS
GDM
Odds_Ratio
Country
0
5
10
15
20
25
